# Marine Reserve Targets to Sustain and Rebuild Unregulated Fisheries

**DOI:** 10.1371/journal.pbio.2000537

**Published:** 2017-01-05

**Authors:** Nils C. Krueck, Gabby N. Ahmadia, Hugh P. Possingham, Cynthia Riginos, Eric A. Treml, Peter J. Mumby

**Affiliations:** 1 Marine Spatial Ecology Lab and Australian Research Council Centre of Excellence for Coral Reef Studies, The University of Queensland, St Lucia Campus, Brisbane, Queensland, Australia; 2 School of Biological Sciences, The University of Queensland, St Lucia Campus, Brisbane, Queensland, Australia; 3 Oceans Program, World Wildlife Fund (WWF), Washington, D. C., United States of America; 4 Australian Research Council Centre of Excellence for Environmental Decisions, The University of Queensland, St Lucia Campus, Brisbane, Queensland, Australia; 5 School of BioSciences, The University of Melbourne, Melbourne, Victoria, Australia

## Abstract

Overfishing threatens the sustainability of coastal marine biodiversity, especially in tropical developing countries. To counter this problem, about 200 governments worldwide have committed to protecting 10%–20% of national coastal marine areas. However, associated impacts on fisheries productivity are unclear and could weaken the food security of hundreds of millions of people who depend on diverse and largely unregulated fishing activities. Here, we present a systematic theoretic analysis of the ability of reserves to rebuild fisheries under such complex conditions, and we identify maximum reserve coverages for biodiversity conservation that do not impair long-term fisheries productivity. Our analysis assumes that fishers have no viable alternative to fishing, such that total fishing effort remains constant (at best). We find that realistic reserve networks, which protect 10%–30% of fished habitats in 1–20 km wide reserves, should benefit the long-term productivity of almost any complex fishery. We discover a “rule of thumb” to safeguard against the long-term catch depletion of particular species: individual reserves should export 30% or more of locally produced larvae to adjacent fishing grounds. Specifically on coral reefs, where fishers tend to overexploit species whose dispersal distances as larvae exceed the home ranges of adults, decisions on the size of reserves needed to meet the 30% larval export rule are unlikely to compromise the protection of resident adults. Even achieving the modest Aichi Target 11 of 10% “effective protection” can then help rebuild depleted catch. However, strictly protecting 20%–30% of fished habitats is unlikely to diminish catch even if overfishing is not yet a problem while providing greater potential for biodiversity conservation and fishery rebuilding if overfishing is substantial. These findings are important because they suggest that doubling or tripling the only globally enforced marine reserve target will benefit biodiversity conservation and higher fisheries productivity where both are most urgently needed.

## Author Summary

Marine reserves are an important tool to conserve biodiversity but increasingly are relied upon also to benefit fisheries, specifically in diverse, unassessed, and otherwise unregulated systems. Whether the globally adopted Aichi Target 11 (10% “effective” protection of national coastal marine areas) can help sustain or rebuild fisheries under such complex conditions is unclear. Based on a systematic theoretic analysis that incorporates previously unavailable data on fish movements, we show that 20%–30% strict protection of fished habitats is unlikely to harm complex and otherwise unregulated fisheries even if most fish populations are still healthy while providing greater potential to rebuild depleted fisheries and protect biodiversity than the 10% Aichi target. This finding is important because it suggests that doubling or tripling the only internationally enforced marine reserve target will support biodiversity conservation and higher fisheries productivity where both are most urgently needed.

## Introduction

Overfishing and other anthropogenic impacts threaten the sustainability of coastal marine biodiversity and ecosystem functioning worldwide [1,2]. To counter this problem, nearly 200 governments have committed to protecting 10% of all coastal and marine areas “effectively” by 2020 (Aichi Target 11 of the Convention on Biological Diversity) [3]. This 10% target for marine reserve coverage supersedes an earlier 20% aspiration and highlights the pervasive conflict between the need to protect biodiversity and human demand for unrestricted access to fishery resources. It is well recognized, for example, that reserves will reduce total fisheries yields where fisheries are managed effectively through other regulations [4]. Thus, many countries with high fisheries management capacity have either been able to restore productive fisheries without implementing reserves [5,6], or they implicitly accept that 10% designation of reserves will support biodiversity while potentially sacrificing some fisheries production.

In much of the developing world, however, food security is a significant concern, in part because fisheries are an essential source of livelihoods but often highly diverse, largely unregulated, and heavily overexploited [6,7,8,9]. In this case, any loss of total fisheries productivity caused by establishing marine reserves for biodiversity conservation that do not offset the catch lost from reserve areas will exacerbate poverty and potentially reduce access to protein. The “Coral Triangle” region of Southeast Asia typifies this challenge, in that the enforcement of marine reserves is seen as critical to fight ongoing biodiversity loss but feasible only if reserves also benefit, or at least do not diminish, adjacent fisheries [10]. Yet, whether the 10% global Aichi target (or the more ambitious 20% long-term reserve coverage goal adopted by member states of the Coral Triangle region [10]) will help sustain and rebuild or induce a net loss in fisheries productivity remains unspecified.

The focus of marine reserve designation for biodiversity conservation is to ensure the persistence of species within reserve boundaries [11]. In contrast, to benefit fisheries, marine reserves must ensure enhanced demographic production and the export of fish as either adults or larvae from reserves to fished areas [12], which is a far more ambitious expectation. Of 57 case studies explicitly analyzing the fishery functioning of reserves, for example, only half found that reserves were actually beneficial and that they should cover 40% ± 20% (mean ± standard deviation [SD]) of fishing grounds to maximize yields or profits [13]. These results and other reviews [14,15] highlight a wide range of potentially suitable reserve coverage policies, which stress the need for a systematic analysis of their expected fishery functioning in order to support management decisions under data-poor conditions.

Here, we use a combination of spatially implicit and spatially explicit models to undertake such a systematic analysis of reserve coverage policy implications. We do this by calculating suitable reserve coverage targets not only for situations in which fisheries are depleted and reserves thus expected to benefit fisheries productivity but also to specify how much fishing ground reserves can maximally cover for biodiversity conservation before initially healthy fisheries become negatively impacted. Importantly, our analysis takes into account the great diversity of and uncertainty about locally variable drivers of fisheries productivity around reserves (Table 1, S1 Table). With complete control over simulations that can integrate most of these drivers, we systematically assess the conditions leading to both desirable and nondesirable reserve impacts when fishers have little or no alternative to fishing (i.e., total fishing effort remains constant at best). In particular, the lack of critical data on fish movements as both adults and larvae across reserve boundaries has thus far hampered reliable predictions of reserve impacts on fisheries [16]. To address this challenge, our models incorporate the latest empirical measurements of adult home ranges and larval dispersal distances on coral reefs [17,18]. We use our findings to advance generic guidance on the interactive role of decisions on reserve size and coverage in order to support data-poor fisheries that are unassessed and otherwise unmanaged. In addition, we revisit the implications of existing (Aichi) and potentially more suitable habitat protection commitments for such vulnerable types of fisheries, whose global importance appears to be underappreciated [8,19].

**Table 1:** Key drivers of the fishery functioning of marine reserves. Arrows highlight the net impact of an increase in parameter value on the maximum reserve coverage for biodiversity conservation without fisheries costs (sustain fisheries) and the optimum reserve coverage to benefit fisheries (rebuild fisheries). Plus signs rank the relative strengths and uncertainties of impacts: + low, ++ medium, +++ strong. The strongest drivers of fishery impacts are marked in bold. See text and S1 Table for explanations.

Parameters	Maximum Coverage to Sustain	Optimum Coverage to Rebuild	Impact/Uncertainty	Tested
Species				
Natural adult mortality	**↑**	**↑**	++/++	Yes
Growth	**↓**	**↓**	+/+	Yes
**Movements**				
Larval dispersal	**↑**	**↓↑**	+++/++	Yes
Juvenile spillover	**↑**	**↓↑**	+++/++	Implicit
Adult spillover	**↑**	**↑**	+++/++	Yes
**Density-dependence**				
Pre-settlement	**↓**	**↑**	+++/+++	Yes (see S1 Table)
Post-settlement				
Intra-cohort	**↓**	**↓**	+++/++	Yes
Inter-cohort	**↓**	**↑**	+++/+++	Yes (see S1 Table)
Inter-specific	**↓↑**	**↓↑**	+++/+++	No [20]
Fishery				
**Exploitation level**	**↓**	**↑**	+++/++	Yes
Effort displacement	**↑**	**↑**	++/++	Yes
Fisher mobility	**↑**	**↓**	++/++	Yes (see S1 Table)
Partial non-compliance	?	↑	?/++	Yes (see S1 Table)
**Catch regulations**	↑	↓	+++/+	No [21,22]
Socio-economic context	↓↑	↓↑	++/+++	Yes (see S1 Table)
Environment				
Stochasticity in recruitment	**↑**	**↑**	++/++	Yes (see S1 Table)
Gradients in habitat quality	**↓↑**	**↓↑**	++/++	Yes (see S1 Table)
Asymmetric connectivity	**↓**	↓	++/+++	Yes (see S1 Table)
**Trophic interactions**	**↓↑**	**↓↑**	+++/++	No [23,24]
Behavioral interactions	**↓↑**	**↓↑**	?/+++	No [25]
Reserve network design				
**Location of reserves**	**↓↑**	**↓↑**	+++/+++	Yes (see S1 Table)
**Size of reserves**	**↓**	**↓**	+++/++	Yes

## Results

We begin with a generic and spatially implicit analysis that represents a great variety of possible biological responses to overfishing intensity by sampling empirical estimates of natural adult mortality, growth, and the density-dependent survival of young fish after settlement. Each scenario in our analysis is representative of a single species and diversified further by varying the capacity for exchange of both adults and larvae between reserves and fished areas. We express this so-called spillover effect across reserve boundaries as a percentage of exchanged adults and larvae, thereby representing various potential scales of adult movements and larval dispersal relative to local reserve sizes.

In all scenarios, we calculate two reserve coverage policy reference points that explicitly distinguish between the pros and cons of reserves for fisheries and biodiversity protection. The first reference point acknowledges that reserves might deplete initially productive fisheries by reducing harvestable area and intensifying unregulated fishing activities in this smaller area [26]. We identify the reserve coverage at which this intensified fishing pressure reaches undesirable levels as catch falling below “pretty good yield” (PGY), which is catch ≥80% of the “maximum sustainable yield” (MSY). Sustaining PGY is a desirable target even if fisheries are well managed [27], while sustaining MSY is massively challenging [28], specifically if fishery conditions are complex [7]. Thus, our first policy reference point can be understood as the “maximum biodiversity benefit without fisheries cost.” Our second reference point recognizes that marine reserves might also rebuild fisheries productivity. It specifies the “maximum fisheries benefit” by quantifying the reserve coverage that optimizes catch increases (if any) relative to pre-reserve conditions.

**Fig 1:**
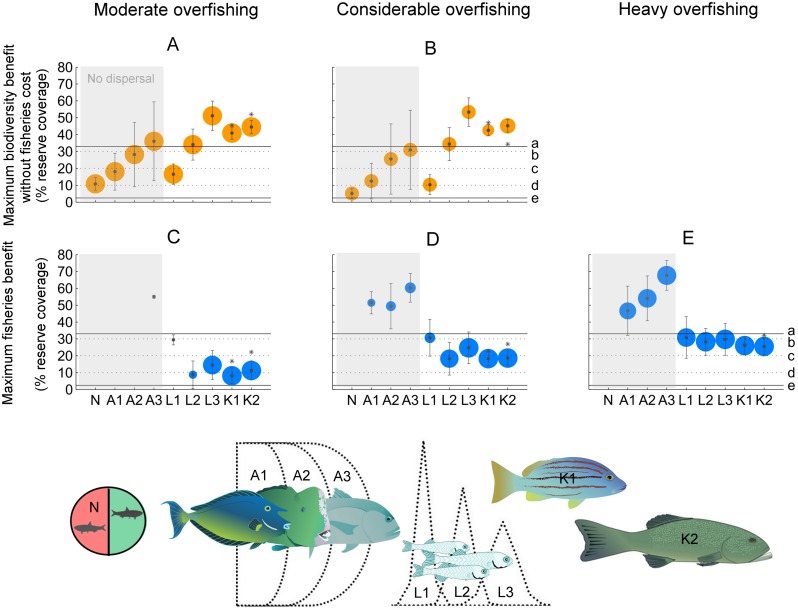
Marine reserve coverage targets for unregulated fisheries under increasing levels of overexploitation. (A–B) The maximum reserve coverage for biodiversity conservation that does not deplete initially good fisheries catch. (C–E) The optimum reserve coverage to rebuild fisheries catch. Circles and errors bars represent means ± SD, scaling in size to the number of fishery scenarios they represent. Small circles, no circles, or no plot mean that the catch was either not initially high (A–B) or not improved through reserves (C–E). Outcomes are categorized based on increasing levels of exchange through adult movements (A1–A3) and larval dispersal (L1–L3) across reserve boundaries. Exchange categories are: (N) no exchange, (1) 10%–20%, (2) 30%–50%, and (3) 60%–100%. Assuming realistic reserve sizes of 1–20 km, the home ranges of most adult coral reef fishes [17] suggest classifications under exchange category A1 or lower (<10%). Dispersal distances of coral reef fish larvae [18,29] suggest classifications under exchange categories L2–L3. Keppel island (K) scenarios represent spatially explicit calculations of exchange for *Lutjanus carponotatus* (K1) and *Plectropomus maculatus* (K2) assuming reserve sizes of 4 ± 4 km (mean ± SD). Results for 2 ± 2 km reserves are indicated by star symbols. The reference lines mark enforced (solid) and anticipated (dotted) reserve coverages: (a) Great Barrier Reef; (b) International Union for Conservation of Nature (IUCN) recommendation; (c) Coral Triangle Initiative (CTI) long-term goal; (d) Aichi and CTI 2020 target; and (e) Coral Triangle. The full range of fishery scenarios included all combinations of fish movements (see all results in S2 Table). See Materials and Methods for details. Images: Catherine Collier (ian-symbol-plectropomus-spp.svg), Christine Thurber (ian-symbol-naso-unicornis.svg), Jane Thomas (ian-symbol-bolbometopon-muricatum.svg, ian-symbol-caranx-ignobilis.svg), and Tracey Saxby (ian-symbol-morone-saxatilis-feeding-larvae.svg), Integration and Application Network, University of Maryland Center for Environmental Science (ian.umces.edu/symbols/); Alice Rogers (*L*. *carponotatus*), University of Queensland.

When overfishing was moderate, which we defined as 1.1 to 1.2 times the rate of annual harvest generating MSY (*F*_MSY_), nearly all fisheries (97%) still delivered PGY. Maximum reserve coverages for biodiversity conservation that did not compromise PGY of these fisheries averaged 54% but were highly variable if larval dispersal was limited (Fig 1A, S2 Table). The fisheries that were most sensitive to reserve implementation (for good or bad) were those targeting fish populations with strong density-dependent mortality of young fish after settlement. Even moderate overexploitation might then deplete fish biomass to 15% or less of unfished levels [27]. Any further biomass depletion caused by fisher concentrations outside of reserves can then result in sharp catch declines (see S1 Fig) unless compensated for by larval dispersal from reserves. However, catch declines below PGY were evident for only 12% of all modelled fishery scenarios even if 20% of the fishing ground was covered by reserves, while nearly half of all fisheries (43%) experienced maximum catch increases at this level of reserve coverage. The key common characteristic of benefiting fisheries was that larval export from reserves was >30% (Fig 1C, S2 Table).

Under considerable overfishing, which we defined as 1.3–1.5 × *F*_MSY_, about half (48%) of all fisheries still delivered PGY without reserves. Possible closures for biodiversity conservation that did not compromise PGY of these fisheries averaged 49%, which was almost identical to predictions under moderate overfishing (Fig 1B, S2 Table). In contrast to the situation under moderate overfishing, catch increases through reserves were then possible for 80% of all fisheries. Specifically when larval export from reserves was ≥30% (scenarios L2–L3), catch increases were not only widespread but also substantial (PGY for >80% of fisheries). Maximum catch increases were evident at reserve coverages of 24% (no spillover of adults from reserves to fished areas) and 43% (all scenarios) (Fig 1D, S2 Table).

Under heavy overfishing, which we defined as 1.6–2 × *F*_MSY_ and which is likely to represent many poorly managed small fisheries worldwide [19], the only feasible fisheries management goal of reserves was to rebuild productivity, because <1% of all fisheries began with PGY, while catch increases were then possible for 98% of all fisheries. If adult fish were fully protected in reserves, reserve coverages that maximized catch increases averaged 30%. If adult spillover reached 60%–100% (i.e., reserves were assumed to be very small relative to adult home ranges), the total reserve coverage had to increase to >50% in order to maximize catch increases (Fig 1E). However, in all scenarios that included realistic levels of the exchange of larvae ≥10%, fisheries productivity was increased, albeit not maximized, even with a relatively low reserve coverage, including that of the 10% Aichi target (S2 Table, S2 Fig).

Across all levels of overfishing intensity, we found very little evidence that achieving realistic reserve coverage targets of up to 30% would risk sustaining PGY in unregulated fisheries. The key to avoiding any such undesirable fishery outcomes will be to ensure that reserve sizes allow for ≥30% export of locally produced larvae to adjacent fishing grounds. In this case, less than 3% of all initially productive fisheries fell below PGY at 30% reserve coverage. Ensuring at the same time that reserves are large enough to protect most resident adults (≥50%) will exclude negative fishery outcomes almost entirely, while making long-term fishery benefits highly likely.

Having obtained these generic insights across a broad parameter space, we then created two species-specific and spatially explicit modelling scenarios that utilized recent measurements of adult home ranges [17] and of larval dispersal distances [18] of the Spanish flag snapper (*Lutjanus carponotatus*) and of the spotted coral grouper (*Plectropomus maculatus*)—two key fishery species in the tropical Pacific. The role of reserves in these species-specific models was consistent with that of the generic version, with the two coral reef fisheries closely aligned to scenarios assuming high to very high exchange of larvae (~50%) and no (snapper) or very low (grouper) exchange of adults (see K scenarios in Fig 1). Under moderate overfishing, our models predicted that up to about 40% reserve area could be designated without impairing the fishery’s ability to maintain PGY (S2 Fig). If the system experienced heavy overfishing with initially depleted catch, reserve coverages required to rebuild catches maximally fell between 20% and 33% (Fig 1, S2 Table, S2 Fig).

## Discussion

Intuitively, our results suggest that 25% no-take reserve coverage could serve as a generic target for rebuilding unregulated fisheries (S1 Fig). However, the underlying assumption is that individual reserves are large enough to protect resident adults while allowing for widespread dispersal of fish larvae to fished areas. Specifically for target species on coral reefs, empirical data now suggest that this assumption is reasonable. Adult movements of most coral reef fishes are restricted to a few kilometers [17] while the first measurements of realized larval dispersal (S3 Fig) [18,29,30] suggest that even 15 km reserves are likely to export 35%–45% of locally produced larvae to adjacent fishing grounds (representing scenario L2 in Fig 1). For scales of adult movements that exceed reserve diameters, however, reserve coverages might have to increase far beyond 25% in order to rebuild fisheries maximally (scenarios A1–A3 in Fig 1).

Coastal fisheries in many tropical regions are suffering from increasing overexploitation, with little or no enforcement of catch regulations [8,31,32,33]. The “Coral Triangle,” as the world’s center of marine biodiversity and conservation priority [34], is a global hotspot of this fisheries crisis [6,9]. Based on our classification of overfishing intensity, coral reef fisheries throughout the “Coral Triangle” are heavily overexploited (at least 1.6–1.7 × MSY) [35]. In this case, achieving the international Aichi target of 10% “effective protection” by 2020 could substantially improve local fisher livelihoods and food security (S2C and S2F Fig). However, at minimally 20%–30% of fishing grounds, optimal reserve coverages to rebuild heavily depleted catch (Fig 1E) are far larger than the Aichi target, and they also surpass the more ambitious long-term Coral Triangle Initiative (CTI) goal of 20% habitat protection [10]. Unfortunately, all of these targets remain ambitious in most of the region; the current “effective” reserve coverage lies between 1%–2% [10], which essentially precludes not only the conservation of biodiversity [11] but also the rebuilding of depleted catch. Achieving higher reserve coverage will be challenging in terms of governance, planning, enforcement, and, critically, the short-term impacts on fishers while fisheries recover. However, recent studies suggest that gradually increasing the number and size of reserves over a period of 10–20 y could help reduce the latter problem by trading off the time taken to achieve future benefits against the short-term cost of losing fishing grounds while fisheries recover [36]. Alternatively, and perhaps counterintuitively, an initially even higher than optimal coverage of reserves would exacerbate immediate costs to fishers but simultaneously help minimize the time required to achieve lasting benefits [37].

An important question to support decisions on the best reserve implementation strategy is how much short-term reduction in catch fishers will be able to tolerate. Answers to this question are intimately linked to local socioeconomic context. All modelling scenarios presented above implicitly assume that the short-term costs of reserve enforcement are bearable or that, as yields decline temporarily, fishers become poorer. They also assume that fishers have no or very limited access to alternative livelihoods, such that target species are exploited until they are no longer available. These assumptions are appropriate to reflect the situation of small-scale fishers in much of the developing world, who are threatened by future food shortage. The dilemma of these fishers in the absence of any form of fishery regulations is a likely mismatch between supply and demand that will ultimately lead to fishery collapse [38]. In consequence, heavy overfishing and an increase in poverty must be considered to represent the fate of any such unregulated fishery.

Our results provide a generic biophysical basis from which to develop reserve coverage policies to rebuild and sustain these types of fisheries. However, the practical implementation of our findings will need to take account of local social and economic states and opportunities. In some regions, fishers will be able to enter or exit fishing activities depending on whether catch is currently profitable [39]. Context-dependent adjustments to models can then be used to refine reserve coverage targets based on their economic impact. Using our two empirically supported coral reef fishery scenarios as examples, we find that suitable reserve coverage targets are unlikely to change even if fishing effort varies dynamically in response to profits and also even if fisheries become unprofitable before fish populations are collapsed (the “stock effect”) [40,41,42]. However, generalizing the implications of socioeconomic context is challenging, as refined reserve coverage targets will depend on interactions among (1) local costs and revenues for a given catch and fishery status, (2) the change in fishing effort (access) for a given change in profit, (3) the duration over which fisheries performance is assessed, and (4) the change in costs and revenue over this time period (see S1 Table and associated figures).

Also from a purely biophysical perspective, it is clear that generic predictions of reserve impacts are complicated by the diversity of potentially important drivers of fisheries productivity. We have listed and explicitly tested many of such potential drivers, and we elaborate on their implications for marine reserve coverage targets in the Materials and Methods and Supporting Information sections. Some potential drivers, which were not incorporated in the results presented above, are either highly uncertain, affect the magnitude of catch increases rather than reserve coverage targets, or have ambiguous implications that cannot be captured by any form of generic decision making. Environmental heterogeneity due to, for example, gradients in habitat quality, can be an important source of positive and negative deviation from inferred marine reserve coverage targets. However, management decisions are unaffected by such heterogeneity unless the placement of reserves is systematic and informed by reliable environmental data.

Similarly strong and ambiguous implications might result from multispecies interactions that generate trophic cascades [43]. Cascades have been observed even on diverse coral reefs when large predators are depleted [44], although theoretic analyses of complex reef fish food webs find them to be stabilized by high levels of omnivory [43]. In general, we expect relatively weak interaction strengths in any ecosystem subjected to substantial overfishing [43]. Given that substantial overfishing is common, specifically where fishery conditions are complex and traditional management tools are hardly available [9], reserves might therefore be expected to facilitate increases in the abundance of both predator and prey species [23]. Implicitly supporting this assumption, meta-analyses of reserve impacts have found empirical evidence for declines in on average 20% of species [45], leaving a great and diverse majority of other species to experience recovery [46,47] and deliver potential catch increases in adjacent fishing grounds.

In the future, robust data might enable locally optimized decisions on marine reserve coverages. However, as yet, even local and spatially realistic fishery models will be unable to capture the full demographic and trophodynamic complexity of real systems, primarily because they could not be parameterized meaningfully for this to be achieved. Simplified generic assessments, as in this study, offer an alternative that exploits the sum of our current empirical understanding. Moreover, such models might be better suited to inform generic decision making. Otherwise, globally adopted marine reserve coverage targets, such as Aichi Target 11, must continue to lack any scientific justification of associated fishery implications.

**Fig 2:**
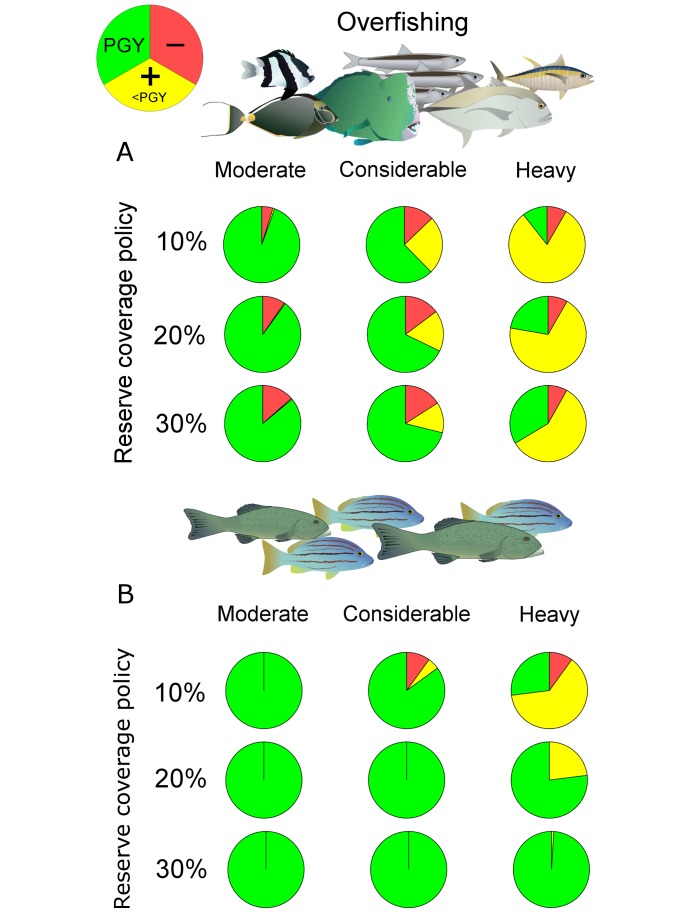
Status and trends of unregulated fisheries under three currently discussed reserve coverage policies. (A) The full range of biological conditions in generic modelling scenarios (*n* = 840,950) captured across a gradient in overfishing intensity. (B) As in A but representing fishery scenarios based on measured reserve sizes, home ranges and larval dispersal distances (*n* = 100) (see K scenarios in Fig 1 and Materials and Methods for details). Green highlights the proportion of healthy fisheries, delivering “pretty good yield” (PGY, ≥80% of the “maximum sustainable yield”) because or in spite of reserves. Red and yellow highlight the proportion of depleted fisheries, delivering less than PGY. While red indicates that reserves decreased catch, yellow indicates that reserves increased catch, albeit not to levels ≥ PGY. All data underlying this figure are available online (10.5281/zenodo.165189). Images: Catherine Collier (ian-symbol-plectropomus-spp.svg), Joanna Woerner (ian-symbol-naso-lituratus.svg), Jane Thomas (ian-symbol-bolbometopon-muricatum.svg, ian-symbol-caranx-ignobilis.svg), Tracey Saxby (ian-symbol-dascyllus-aruanus.svg, ian-symbol-thunnus-albacares.svg), and Dieter Tracey (ian-symbol-engraulis-australis.svg), Integration and Application Network, University of Maryland Center for Environmental Science (ian.umces.edu/symbols/); Alice Rogers (*L*. *carponotatus*), University of Queensland.

It has long been argued that 30% or more reserve coverage of a species’ habitat is needed to maintain its persistence and protect marine biodiversity [11,14]. Our study suggests that a similar argument can be made for sustaining or rebuilding complex and otherwise unregulated fisheries, despite the more ambitious ecological challenge of sustaining or enhancing population productivity in nonreserve areas [12]. This is important because it implies that biodiversity conservation and fisheries objectives are generally compatible in many heavily exploited parts of the world that lack conventional fisheries regulations. While some exceptions exist in which achieving any of the internationally enforced reserve coverages between 10% and 30% can deplete unregulated fisheries (Fig 2A), such situations were rare, accounting for less than 10% of all modelled fishery conditions (*n* = 840,950). Such detrimental reserve impacts were mostly confined to fisheries with limited export of larvae across reserve boundaries. Once reserves exported ≥30% of larvae, only 3% of modeled scenarios resulted in deleterious fisheries impacts.

Importantly, the degree of export from reserves and, thus, the likelihood of any negative fishery outcomes can be influenced by adapting local reserve sizes to the estimated scale of larval dispersal and adult movements. For typical fishery species on coral reefs like the Spanish flag snapper and spotted coral grouper—some of the most important in the Pacific—we found that achieving any reserve coverage policy target between 20% and 30% would allow PGY to be sustained or catches to increase (Fig 2B) provided that the mean diameter of individual reserves was kept below a conservative estimate of 15–20 km (approximately double the mean larval dispersal distance). Then, even the modest Aichi Target 11 of 10% “effective” protection of fished habitat provided some fisheries benefit without compromising long-term fisheries productivity. However, higher reserve coverages of 30% provided for optimum rebuilding of overexploited fisheries while still not adversely impacting productivity in potentially healthy fisheries. If adults are more mobile than measured on coral reefs, then fisheries catch might be largely insensitive to reserve enforcement or require much higher reserve coverages for optimum rebuilding. Thus, from a generic biophysical standpoint, 20%–30% reserve coverage appears to serve as a safer generic target than 10% in order to meet both biodiversity conservation and fisheries objectives in unassessed and otherwise unregulated ecosystems.

## Materials and Methods

### Modelling procedure

Spatially implicit modelling scenarios were based on an annual time step, starting each year with a spawning event, followed by larval dispersal, settlement, and the subsequent recruitment of fish in protected and fished areas. Local larval output was recalculated each year based on the amount of fish biomass in each of these two management zones. Exchange between management zones was introduced based on the larval dispersal parameter *d* and the adult movement parameter *m*. Both parameters represented a proportion of complete bidirectional exchange across reserve boundaries. For example, a value of *d* = 0.1 resulted in 10% of larvae from each area being redistributed to protected and fished areas according to their relative proportions of the seascape. Ultimately, for *d* = 1 and *m* = 1, all larvae and adults were distributed evenly across the seascape.

Recruitment of larvae (*L*) at a location *i* and time *t* was calculated based on the Beverton–Holt function with steepness (Eq 1):

$$R_{i,t}=\;\frac{L_{i,t}}{\alpha +\beta L_{i,t}},\text{ with }\alpha =\;\frac{L_{0,i}}{R_{0,i}}-\beta L_{0,\text{i}},\text{and }\beta =\;\frac{5h-1}{4hR_{0,i}}\;,$$ (1)

where *R* is the number of larvae surviving to recruitment age calculated based on constants *α* and *β*, which define the initial slope and asymptote of the recruitment curve. The steepness parameter *h* specifies the degree of intracohort density-dependent mortality by defining the proportion of young fish surviving to recruitment age when larval supply equals 20% of natural (i.e., unfished) larval settlement (*L*_0_). Thus, recruitment was nearly identical to larval supply when *h* ≈ 0.2 and almost constant when *h* ≈ 1 (S1 Fig).

Biomass (*B*) production dynamics were calculated subsequent to recruitment based on a Deriso-Schnute delay-difference algorithm [48] (Eq 2):

$$B_{i,t}=s_{t-1}\;B_{i,t-1}\;+\;p\;s_{t-1}B_{i,t-1}\;-\;p\;s_{t-1}\;s_{t-2}B_{i,t-2}-p\;s_{t-1}\;w_{L-1}R_{i,t-1}+\;w_{L}R_{i,t},$$ (2)

where *s* is the annual survival of adults, *p* is the Brody growth coefficient, and *w*_*L*_ and *w*_*L-1*_ are fixed weights of fish at and prior to recruitment, respectively. Without explicitly simulating age structure, which is generally unknown for target species in unassessed and unregulated fisheries, the delay-difference model enables simulations of biomass production dynamics that incorporate principal time delays in growth and recruitment parameterized according to empirical measurements [49]. The predictive capacity of delay-difference models is comparable to much more complex ecological models [50].

Fish biomass in fished areas was harvested according to the fisheries mortality rate *F* (multiples of *F*_MSY_). Total fishing effort was assumed to be constant (all fishers kept fishing) by adapting the fishing mortality rate at fishing grounds according to the current level of reserve coverage (*C*): *F’* = *F*/(1 − *C*) [26]. In all scenarios, the modelling procedure was repeated until equilibrium conditions were achieved.

The modelling procedure for spatially explicit reef fishery scenarios was based on the same calculations and sequence of events as described above but using a modelling environment composed of a two-dimensional grid that enabled dynamic simulations of both fish and fisher movements across a heterogeneous seascape. By default, our simulations assumed a homogenous 100 km coastline in 100 m resolution (1,000 grid cells). The distribution of adults and larvae was calculated by fitting a Gaussian distance decay function to measured means and associated SDs of adult home ranges and realized larval dispersal distances. The resulting model predictions yielded a close match to field observations (see S3 Fig).

The size and spacing of reserves was determined by sampling at random from the negative binomial distribution, but sampling probabilities were set so as to yield prespecified means and SDs. To achieve different levels of reserve coverage (*C* = 0%–100%) while holding the mean size of reserves (*S*) constant, the mean spacing between reserves (*O*) was adjusted as follows: *O* = *S*(1 –*C*)*C*^*-*1^. The coefficient of variation of *O* was normalized to 1, resulting in a high level of random variation in spacing between reserves and across repeat runs. Total fishing pressure was kept constant, with values of *F* = 1 per cell normalized again to delivering MSY.

### Parameterization

The mortality and growth of adults in generic scenarios were sampled as value pairs from an empirical dataset on 175 fish stocks [51]. The most likely range to sample was narrowed down to 18%–88% mortality per year, but neither a narrower nor a wider range affected our conclusions. Density-dependent mortality after settlement was narrowed down to steepness values of 0.5–0.9, representing a mean ± SD of 0.7 ± 0.2 as inferred by meta-analysis from observed relationships between fish stock size and recruitment (S1 Fig) [52]. The exchange of both adults and larvae across reserve boundaries was parameterized to explore the full parameter space of our model.

Spatially explicit scenarios for *L*. *carponotatus* and *P*. *maculatus* were parameterized by using estimates of natural adult mortality and growth available from Fishbase [53]. Field measurements of adult home ranges [17] and realized larval dispersal distances [18] were used to calculate the distribution of both adults and larvae (S3 Fig). Home range measurements for *P*. *maculatus* were unavailable, but averaged means and SDs for two congeners (*P*. *areolatus* and *P*. *leopardus*) [17] provided a close match to field measurements [18]. By default, all scenarios assumed reserve sizes of 4 ± 4 km (mean ± SD), representing calculations for the Keppel Islands section of the Great Barrier Reef Marine Park where all larval dispersal measurements were taken. Various other reserve sizes were used to quantify associated implications, including an assumed global average of 2 km [47]. The placement of reserves was allocated at random unless otherwise specified.

In order to incorporate scenarios for *L*. *carponotatus* and *P*. *maculatus* into our spatially implicit analysis as presented in Figs 1 and 2, we used the spatially explicit modelling environment to calculate the mean exchange of adult biomass and numbers of larvae between reserves and fished areas (S4 Fig). Means were based on 100 randomly designed reserve networks for each level of reserve coverage. A summary of parameter values for all scenarios is given in S3 Table.

#### Details on management reference points

Our first management reference point for reserve coverage policy decisions (the “maximum biodiversity benefit without fisheries costs”) was based on the assumption that even regularly assessed and controlled fisheries in developed countries are difficult to maintain close to MSY or maximum economic yield (MEY) [28,54]. For most near-shore fisheries in the tropics, this is likely to be an “unattainable dream” [7]. Keeping MSY as a theoretical baseline, we therefore used a less stringent metric to define high capture productivity of fisheries. Following Hilborn [27], we referred to this metric as PGY (i.e., catch ≥80% of the MSY). We assumed that any unregulated fishery delivering PGY is in a very good state, specifically if reserves are in place to help sustain and stabilize this high level of capture productivity into the future. However, if initially good catch adjacent to reserves starts falling below PGY, the total fish biomass supporting a fishery may be declining below internationally enforced targets and limits for stock recovery, including (1) the World Summit and United Nations Rio+20 Conference on Sustainable Development stock recovery targets (fish biomass ≥ *B*_MSY_, i.e., ~15%–50% of unfished *B*), (2) directives of the European Union defining the same recovery target (fish biomass ≥ *B*_MSY_), and (3) national harvest policies, such as in Australia and New Zealand, which define a limit biomass of 20% of unfished *B* to initiate rebuilding programs [55]. We considered reserve coverages that cause catch to fall below PGY as undesirable, and our first management reference point specifies a potential limit for this negative fishery impact. We acknowledge that decision makers may choose to stay well below this limit.

Our second management reference point for the rebuilding of capture productivity (the “maximum fisheries benefit”) was formulated as a management target, specifying the optimum reserve coverage needed to maximize catch relative to pre-reserve conditions. This reference point only applied if reserves actually increased catch.

#### Density-dependence assumption in main scenarios

Based on a meta-analysis of the relationship between spawning stocks (or numbers of spawned eggs) and recruitment [52], we assumed a likely range of postsettlement density-dependent mortality corresponding to steepness values *h* of 0.5–0.9 (mean ± SD = 0.7 ± 0.2). This parameterization assumes high recruitment even if larval supply is low (by definition of *h*, a value of 0.7 results in 70% of maximum recruitment even if only 20% of the natural number of fish larvae settle) (S1 Fig). We acknowledge that some fishery species are more vulnerable, requiring higher critical replacement thresholds that correspond to values of *h* around 0.4 [11]. However, typical fishery species are the focus of this study, and such species are likely to show high recruitment compensation [52,56]. Trying to conserve or rebuild all fisheries, including those targeting species that are most sensitive to larval supply, would require higher reserve coverage targets (S1 Table). This is a commendable goal but unlikely to reflect the fisheries management focus under conditions involving overexploitation and potential food shortage. We also acknowledge that other forms of density dependence exist, and we elaborate on associated implications and uncertainties in S1 Table.

### Additional modelling scenarios and parameterization

Additional modeling scenarios were run to include recruitment stochasticity, fisher concentration along the edges of reserves (“fishing the line” behavior), partial noncompliance, presettlement density dependence, intercohort postsettlement density dependence (overcompensation), gradients in habitat quality, asymmetric dispersal of larvae by ocean currents, systematic marine reserve placement, and local socioeconomic context. We started by including the first five of these potential drivers of fisheries productivity, because they were insensitive to systematic reserve placement. Stochasticity was included globally by reducing total annual recruitment based on a randomly drawn mortality factor between 0 and 1. Fisher movements were introduced based on a method by Hilborn and colleagues [21], which allowed us to modify the tendency of fishers to concentrate in regions of highest fish biomass. A fisher movement value of 1 resulted in the “ideal free distribution” (IFD) [57], while higher fisher movement values resulted in increasingly extreme “fishing the line” behavior (S5 Fig) [21]. Partial noncompliance was incorporated by introducing poaching events that reduced the overall recovery potential in reserves by 50%, resulting in a maximum of 50% of unfished biomass in reserves. Presettlement density dependence was incorporated by introducing mortality of larvae with a dependence on intracohort size both prior to (global) and after settlement (local) and by assuming that both processes are equally important. Postsettlement density dependence with a dependence on adult densities (overcompensation) was introduced based on Ricker’s recruitment function (Eq 3):

$$R_{i,t}=\;L_{i,t}e^{\alpha -\beta L_{i,t}},\text{ with }\alpha =\text{ln}\left(\frac{R_{0,i}}{L_{0,i}}\right)+\beta L_{0,i},\text{and }\beta =\;\frac{\text{ln}\left(5h-1\right)}{0.8\;L_{0,i}}\;.$$ (3)

Steepness *h* was parameterized as shown in S3 Table (default = 0.7), resulting in minor overcompensation, which appears to adequately represent most fish populations for which the Ricker function provides a better fit than the Beverton-Holt function [56]. Isolated implications of all five of these potential drivers of fisheries productivity are explained in S1 Table and illustrated in S6 Fig.

We continued by quantifying the implications of strong gradients in habitat quality and asymmetric larval connectivity under alternative reserve placement strategies. Habitat quality gradients were introduced by random modification of local carrying capacities (*B*_0_). The magnitude of variation was 100%, i.e., any proportion of local maxima (*B*_0_ = 1). We then applied three different strategies for reserve placement: (1) random reserve selection (ad hoc or mixed strategy under limited environmental information), (2) a systematic selection of highest-quality habitat in reserves, and (3) a systematic selection of lowest-quality habitat in reserves. Intuitively, the latter two strategies resulted in opposite extremes of deviation (~20%) from the generic reserve coverage targets inferred based on random reserve placements (S7 Fig).

Asymmetric connectivity was introduced firstly by modifying the baseline scenario such that larval dispersal was restricted to a single flow direction. We then introduced more realistic levels of asymmetry by accessing a dataset available from dispersal simulations of coral trout (*P*. *leopardus*) larvae across 425 coral reef areas in the “Coral Triangle” region [58]. Three subsets of this dataset, each consisting of 50 neighboring coral reef areas with variable connectivity characteristics, were extracted as exemplary dispersal patterns for this study: (1) “advective,” (2) “patchy,” and (3) “hotspots.” The “advective” dispersal pattern showed the maximum observed sum of differences between upstream and downstream dispersal among reef areas. The “patchy” dispersal pattern showed the maximum observed number of completely isolated reef areas. The “hotspots” dispersal pattern showed the maximum observed SD in dispersal among reef areas. Similar to our habitat quality gradient analysis, we then simulated fishery outcomes under three different reserve siting strategies, using (1) a random reserve selection as our “ad hoc” strategy (or “mixed” strategy under limited environmental information). Presuming perfect knowledge of the system, the two alternative strategies we applied were to (2) select reserve locations with the highest export of larvae to other locations and to (3) select reserve locations with the highest levels of local larval retention. Again, the random reserve site selection strategy was found to have little impact on the validity of generic reserve coverage targets. Intuitively, however, reserves will have limited capacity to sustain or rebuild fisheries if connectivity is very low (the “isolated” dispersal pattern). Our results also confirmed that if reserve placement can be based on robust connectivity information, fishery outcomes can be substantially improved (S8 Fig).

For the final set of additional scenarios, we modified our models to assess the implications of socioeconomic context for decisions on reserve coverage targets. For convenience, we modified the spatially implicit version of our model, given that this simpler model resembled outcomes for both empirically supported species scenarios (*L*. *carponotatus* and *P*. *maculatus*) very closely. We started by defining profit (*P*) as: *P* = *Y*–*Eθ*, where *Y* is yield (the revenue), *E* is fishing effort (the cost), and *θ* is a parameter to adjust at which level of biomass depletion fishing becomes unprofitable [42]. We expressed *Y* and *E* in the same (arbitrary) units and parameterized *θ* such that values of 1 replicated our default assumption (fishes are exploited profitably until they are no longer available). Values of *θ* > 1 introduced a profitability threshold or “stock effect” [59]. For small-scale fisheries, the costs of fishing are likely to be low, resulting in a low profitability threshold, such as, for example, 5% of virgin biomass [36]. However, we analyzed a wider range of possible thresholds up to 20% virgin biomass in order to explore associated implications for reserve coverage targets (S9 Fig).

Open access dynamics were incorporated into our model by letting the fishing effort respond myopically to fishery profits [39]: *E*_*t*+1_ = *E*_*t*_*δ*(*P*_*t*-1_, *P*_*t*_, *dE*_max_), where factor *δ* is a function of fishery profits in the previous and current year (*P*_*t*-1_ and *P*_*t*_) as well as of the maximum relative change in fishing effort (*dE*_max_) a fishery might experience after two consecutive years of gains or losses in profits. For example, if fishery profits doubled, and *dE*_max_ was set to 100%, then fishing effort in year *t* + 1 was doubled. If profits halved, then fishing effort was halved. Values of *dE*_max_ = 0 replicated our default assumption of constant fishing effort (S9 Fig). In addition to this mode of behavior, we investigated the implications of more aggressive resource exploitation. More aggressive fisher behavior was incorporated by reducing effort only if costs exceeded revenues (negative profits), resulting in fishery collapse for much lower values of *dE*_max_ and a general need for reserves in order to sustain or recover profits.

As in previous scenarios, our bioeconomic analysis was focused on the long-term equilibrium of fishery performance, implicitly assuming no discounting. However, the value of fish and the costs of fishing are linked to the time horizon over which fishery profits are assessed. To analyse the implications of the future value of money, we therefore included a discounting term into profit calculations for a subset of realistic short-term scenarios: *P* = *Y*–*Eθ*/(1 + *D*)^*t*^, where *D* is the annual discount rate (S10 Fig) [36,37]. Typically, values of 0.1 (10%) are used to parameterize *D* over short time periods (e.g., 20 y), but in small-scale subsistence fisheries, *D* might exceed 0.2 because artisanal fishers are highly dependent upon fishing and often forced to maintain fishing activities at unsustainable levels [37,60].

## Supporting Information

S1 Fig The influence of post-settlement density-dependent mortality on recruitment calculated based on the Beverton-Holt function. The “steepness” parameter h, which quantifies the degree of recruitment compensation, is a traditional uncertainty in fishery models with a critical impact on predictions of recruitment (A) and, thus, sustainable fishery yield (B). High recruitment compensation, which is equivalent to an average steepness value of 0.7, must be assumed to represent most fish stocks [52], indicating maximum surplus recruitment at only 25% of the unfished biomass or natural settlement of larvae (*L*_0_). Depletions below 25% *L*_0_ can result in sharp catch declines. *R*_0_, unfished or maximum recruitment.
(PNG)

S2 Fig Spatially-explicit predictions of the impact of reserve coverage on the percentage of unfished biomass and maximum catch for two key fishery species. Outcomes represent the Spanish flag snapper *Lutjanus carponotatus* (A-C) and the spotted coral grouper *Plectropomus maculatus* (D-F). Lines are medians ± ranges across 100 random reserve network designs that assumed reserve sizes of 4 ± 4 km (mean ± SD). Overfishing intensity, in units of annual harvest rates delivering the maximum sustainable yield, was 1.2 for moderate, 1.5 for considerable, and 1.8 for heavy. The dashed grey lines highlight fish biomass and fishery catch if the Aichi Target 11 of 10% effective protection was achieved. Images: Catherine Collier (ian-symbol-plectropomus-spp.svg), Integration and Application Network, University of Maryland Center for Environmental Science (ian.umces.edu/symbols/); Alice Rogers (*L*. *carponotatus*), University of Queensland.
(PNG)

S3 Fig Larval dispersal kernels used for all spatially-explicit calculations. Kernels (solid lines) are based on the Gaussian distance decay function fitted to mean (±SD) realized dispersal distances of (A) the Spanish flag snapper *Lutjanus carponotatus* (7.4 ± 8.5 km) and (B) the spotted coral grouper *Plectropomus maculatus* (8.6 ± 7.5 km). Model predictions matched field measurements (grey bars) on the Great Barrier Reef [18], yielding an intentionally conservative fit at high dispersal distances, because the demographic implications of realized dispersal remain uncertain. However, studies are beginning to show that the dispersal potential of many species is substantially higher than predicted here [29, 61], including that of *P*. *maculatus*. These recent data highlight that the capacity of reserves to benefit fisheries estimated in this study is potentially conservative.
(PNG)

S4 Fig Exchange of fish across reserves boundaries calculated based on measured home ranges of adults and dispersal distances of larvae for the Spanish flag snapper *Lutjanus carponotatus* (A-B) and the spotted coral grouper *Plectropomus maculatus* (C-D). The results represent means across 100 random reserve network designs. Plots on the left assume reserve sizes of 4 ± 4 km (mean ± SD). Plots on the right assume reserve sizes of 2 ± 2 km. The values shown here were used as input for spatially-implicit Keppel island (K) scenarios presented in Figs 1 and 2. Sharp increases in the exchange of adults from fished areas to reserves at high levels of reserve coverage (blue dashed lines) reflected that fewer fish will be exposed to fishing even if the center of their home range is located in a fished area. Sharp declines in exchange at the highest reserve coverages in B and D result from the reserve design procedure, which forcedly switched from being able to represent multiple separate reserves to having either two or a single large reserve.
(PNG)

S5 Fig Distribution of fishers (A), catch (B), and catch per unit of effort (C) in relation to the fisher movement parameter used in additional simulations. Results are based on our spatially-explicit model for the snapper (*Lutjanus carponotatus*, see S3 Table for parameterization) and mean reserve sizes of 1-2 km, presenting a snapshot of equilibrium fishery conditions after the enforcement of reserves that cover 25% of an overfished fishing ground (1.5 × *F*_MSY_). Setting the fisher movement parameter to 0 resulted in stationary fishing activities and unevenly distributed catch increases per fisher. A fisher movement parameter of 1 resembled an ideal free fisher distribution (catch per fisher is spatially uniform), while a higher value of 3 caused fisher concentrations along the edges of reserves that reversed the distribution of inequality in catch per fisher observed under stationary fishing.
(PNG)

S6 Fig Isolated impacts of five potential drivers of fisheries productivity on reserve coverage targets. Results show declines in catch under optimal exploitation (blue), and increases in catch under over-exploitation (red) as predicted by our spatially-explicit model in 1 km resolution. All scenarios represent the Spanish flag snapper (*Lutjanus carponotatus*) and mean reserve sizes of 1–2 km. The baseline scenario (A) is otherwise based on the default parameterization given in S3 Table. In (B) stochasticity in annual recruitment levels is incorporated (0–100%). In (C) fishers concentrate along the edges of reserves (fisher movement parameter = 3). In (D) illegal poaching reduces the recovery potential in reserves by 50%. In (E) density-dependent mortality prior to and after the settlement of larvae is equally important. In (F) Ricker’s recruitment function is used to incorporate minor overcompensation (i.e. reduced recruitment when fish biomass approaches unfished levels). See S1 Table and Materials and Methods for details.
(PNG)

S7 Fig The impact of strong gradients in habitat quality and systematic reserve design on coverage targets. Results refer to the maximum reserve coverage without fisheries costs (A) and the optimum reserve coverage for fishery rebuilding (B) under different reserve siting strategies. The dashed line in A indicates the maximum coverage for our baseline scenario under initially optimal exploitation (*Lutjanus carponotatus*, see S3 Table). In B, the same conditions are shown but assuming considerable over-exploitation, and with the dashed line referencing the optimum reserve coverage for rebuilding. Ad-hoc (random) reserve siting was found to be unlikely to change management targets, but systematic reserve placement in regions of either lowest or highest habitat quality would.
(PNG)

S8 Fig The impact of large-scale asymmetric connectivity and systematic reserve design on coverage targets. Results refer to the maximum reserve coverage without fisheries costs (left) and the optimum reserve coverage for fisheries rebuilding (right) under different reserve siting strategies. Dashed lines in plots reference reserve coverage targets for our baseline scenario (*Lutjanus carponotatus*, see S3 Table). In A and B, outcomes for the baseline scenario are compared to a scenario that assumes the same dispersal distances but that dispersal is unidirectional. In C-H, three examples of asymmetric connectivity derived from biophysical simulations of larval dispersal by ocean currents were used. Asymmetric connectivity tends to reduce catch, but not necessarily coverage targets (see D), under ad-hoc reserve site selections. Systematic reserve siting can improve fishery outcomes (E-H). See Materials and Methods for details.
(PNG)

S9 Fig Reserve impacts on long-term fishery profits under open access dynamics. Upper plots assume increasing levels of change in fishing effort relative to current profit under initially optimal exploitation (A) and overexploitation (B). Maximum biannual change in effort ≥50% resulted in fishery collapse if no reserves were implemented. Lower plots (C) and (D) assume maximum changes in fishing effort of 10% (red in A and B) for increasing levels of fishing costs represented by the profitability threshold or “stock effect”. Again, plots to the left and right assume initially optimal exploitation (C) and overexploitation (D). All results are fishery profits 100 years after reserve enforcement. The dashed vertical lines reference our generic biophysical reserve coverage target. MEY, Maximum Economic Yield. See Materials and Methods for details.
(PNG)

S10 Fig Reserve impacts on short-term fishery profits under open access dynamics. Results are based on the biological assumptions specified for the snapper *Lutjanus carponotatus* in S3 Table, showing an initially healthy (A) and overfished (B) fishery. Simulations assume (1) dynamic changes in effort (10% maximum biannual change), (2) a profitability threshold (5% of virgin biomass), and (3) variable discount rates. The outcomes are fishery profits 10 years after reserve enforcement. The dashed vertical lines reference our generic biophysical reserve coverage target. MEY, Maximum Economic Yield. See Materials and Methods for details.
(PNG)

S1 Table Key drivers of the fishery functioning of reserves. This table is an annotated version of Table 1, providing a description of mechanisms behind simulated fishery trends, as well as additional information on parameter impacts and uncertainties.
(DOCX)

S2 Table Marine reserve coverage targets to sustain and rebuild unregulated fisheries. Values are means ± standard deviations as presented in Fig 1, but showing results for all combinations of fish movement scenarios. Percentages of catch and biomass refer to maxima, i.e. maximum sustainable yield and unfished biomass. Minimum and maximum reserve coverages under variable levels of overfishing intensity are highlighted in bold.
(DOCX)

S3 Table Parameters and parameterization used to derive marine reserve coverage targets. The numbers in brackets denote default assumptions (if any) and samples sizes presented in Fig 1. The two Keppel islands scenarios were run using both the spatially-implicit and spatially-explicit version of our model.
(DOCX)

## Abbreviations

CTI: Coral Triangle Initiative
IFD: ideal free distribution
IUCN: International Union for Conservation of Nature
MEY: maximum economic yield
MSY: maximum sustainable yield
PGY: pretty good yield
SD: standard deviation
