# Supplementary figures and images for: Marine Reserve Targets to Sustain and Rebuild Unregulated Fisheries
Source: PLoS Biol. 2017 Jan 5;15(1):e2000537. doi: 10.1371/journal.pbio.2000537 (PMC5215937; doi:10.1371/journal.pbio.2000537)

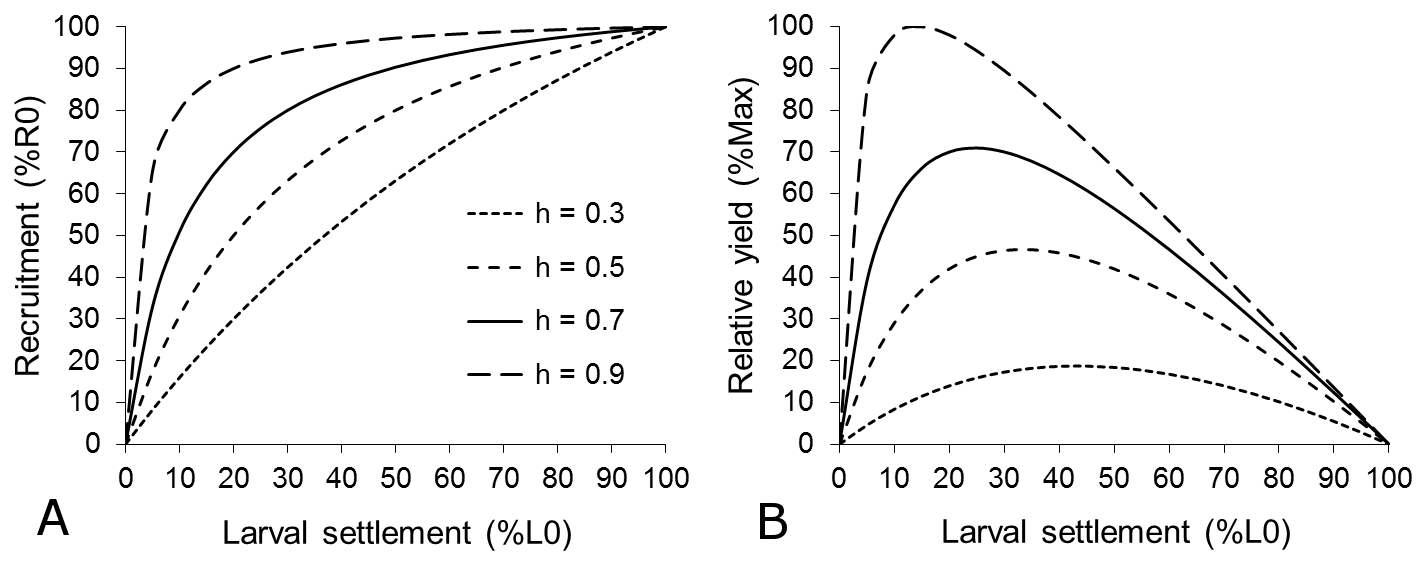

Supplement: S1 Fig — The “steepness” parameter h, which quantifies the degree of recruitment compensation, is a traditional uncertainty in fishery models with a critical impact on predictions of recruitment (A) and, thus, sustainable fishery yield (B). High recruitment compensation, which is equivalent to an average steepness value of 0.7, must be assumed to represent most fish stocks [52], indicating maximum surplus recruitment at only 25% of the unfished biomass or natural settlement of larvae (L0). Depletions below 25% L0 can result in sharp catch declines. R0, unfished or maximum recruitment. (PNG) [file pbio.2000537.s001.png]

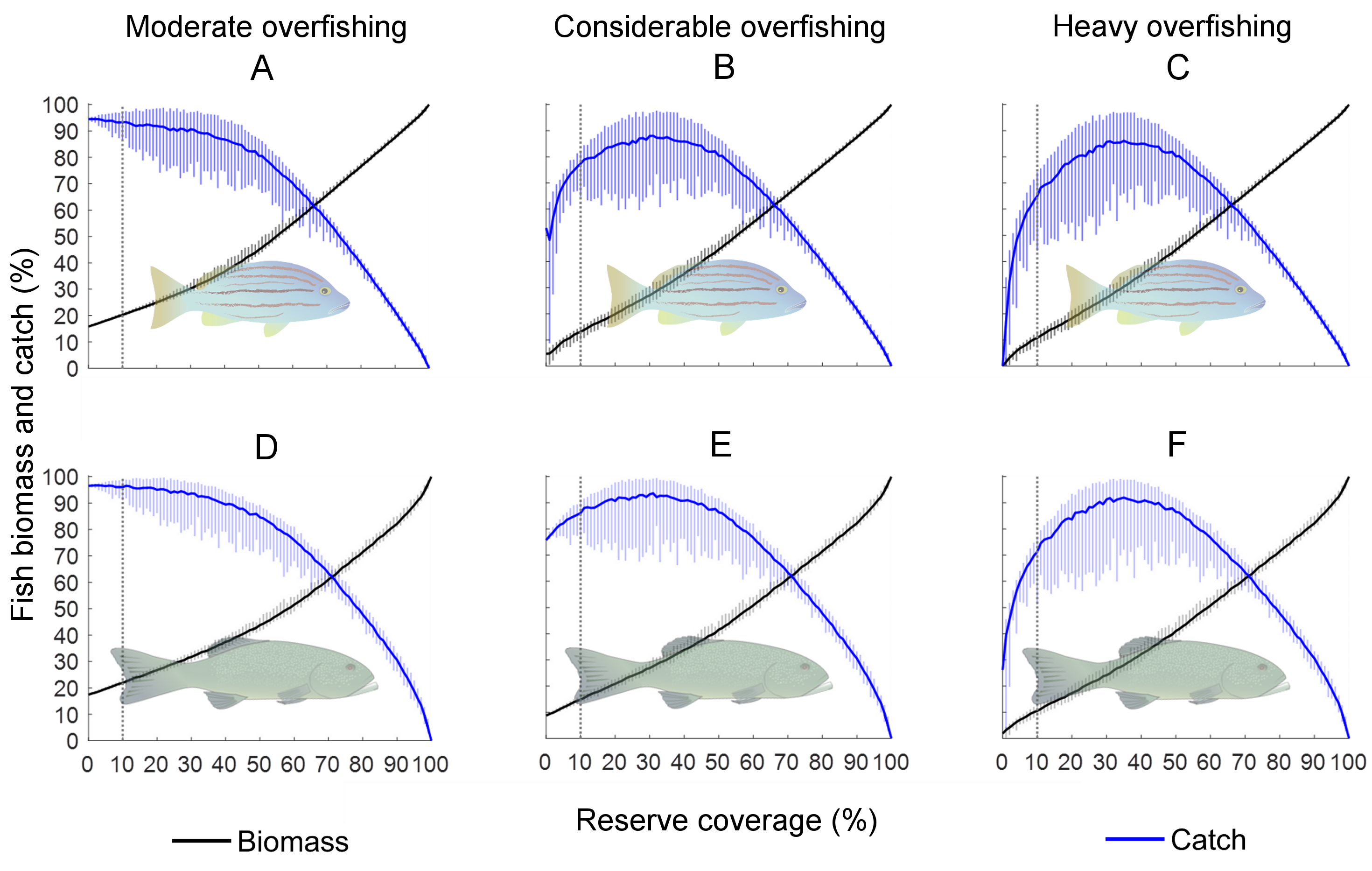

Supplement: S2 Fig — Outcomes represent the Spanish flag snapper Lutjanus carponotatus (A-C) and the spotted coral grouper Plectropomus maculatus (D-F). Lines are medians ± ranges across 100 random reserve network designs that assumed reserve sizes of 4 ± 4 km (mean ± SD). Overfishing intensity, in units of annual harvest rates delivering the maximum sustainable yield, was 1.2 for moderate, 1.5 for considerable, and 1.8 for heavy. The dashed grey lines highlight fish biomass and fishery catch if the Aichi Target 11 of 10% effective protection was achieved. Images: Catherine Collier (ian-symbol-plectropomus-spp.svg), Integration and Application Network, University of Maryland Center for Environmental Science (ian.umces.edu/symbols/); Alice Rogers (L. carponotatus), University of Queensland. (PNG) [file pbio.2000537.s002.png]

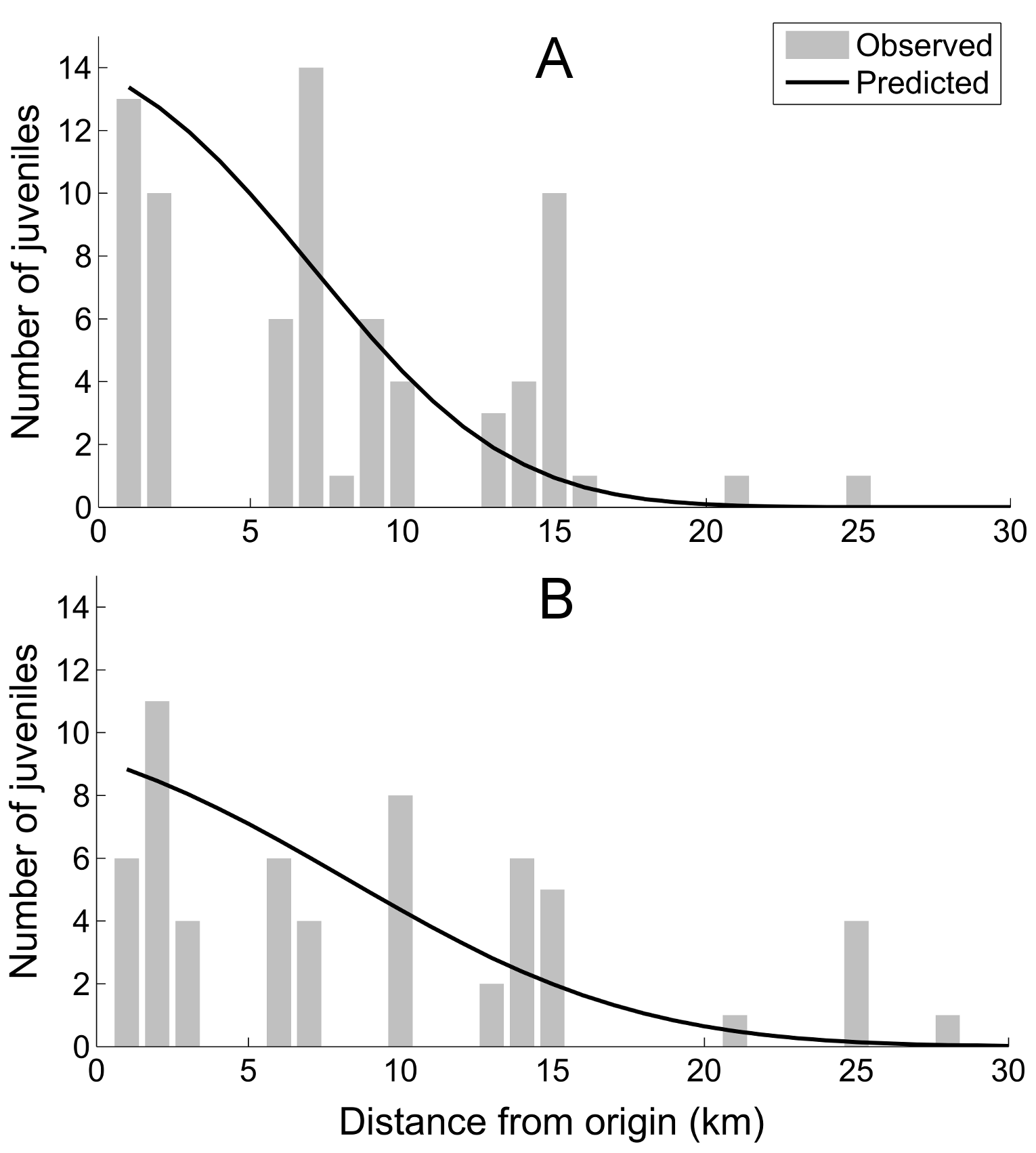

Supplement: S3 Fig — Kernels (solid lines) are based on the Gaussian distance decay function fitted to mean (±SD) realized dispersal distances of (A) the Spanish flag snapper Lutjanus carponotatus (7.4 ± 8.5 km) and (B) the spotted coral grouper Plectropomus maculatus (8.6 ± 7.5 km). Model predictions matched field measurements (grey bars) on the Great Barrier Reef [18], yielding an intentionally conservative fit at high dispersal distances, because the demographic implications of realized dispersal remain uncertain. However, studies are beginning to show that the dispersal potential of many species is substantially higher than predicted here [29, 61], including that of P. maculatus. These recent data highlight that the capacity of reserves to benefit fisheries estimated in this study is potentially conservative. (PNG) [file pbio.2000537.s003.png]

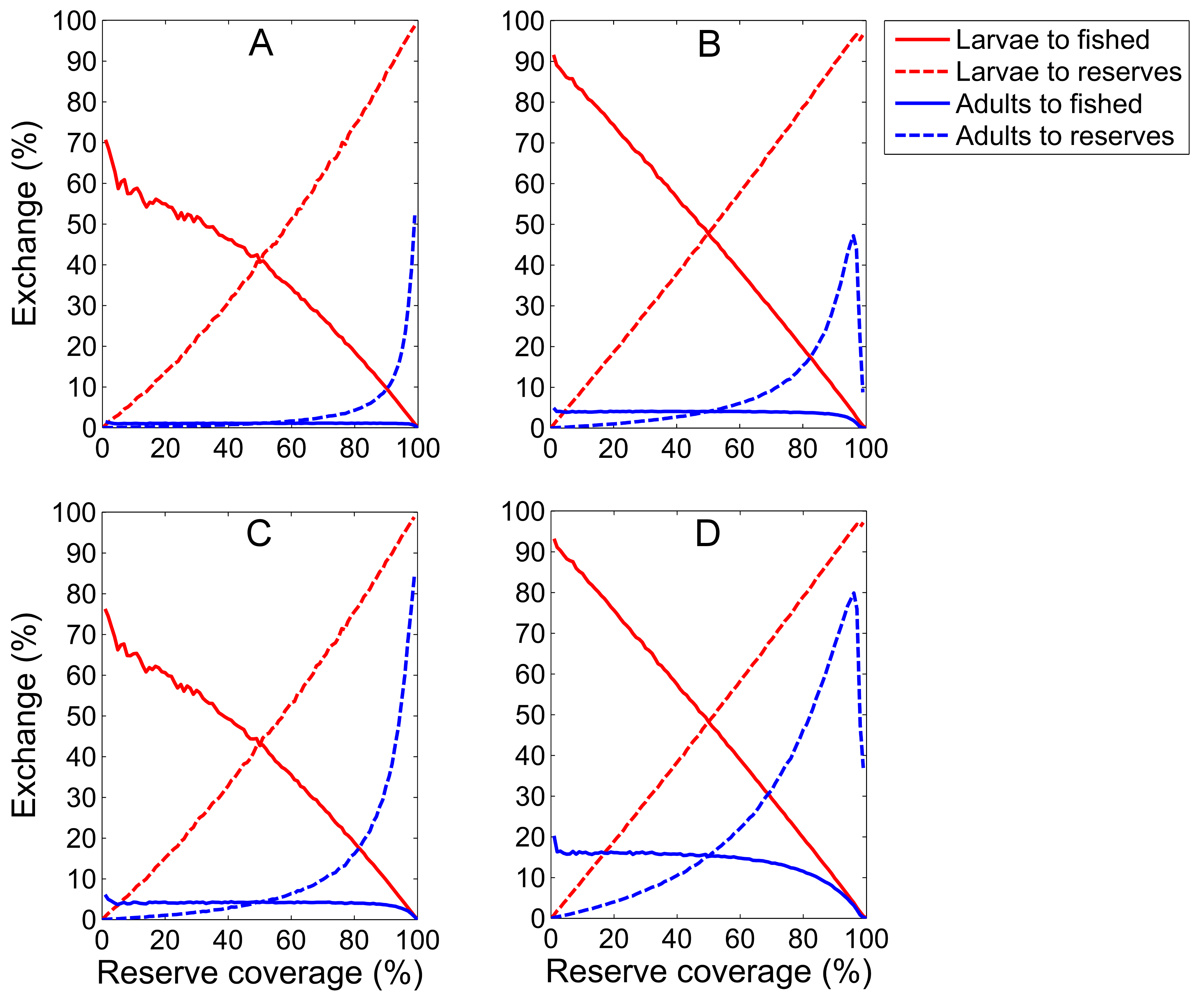

Supplement: S4 Fig — The results represent means across 100 random reserve network designs. Plots on the left assume reserve sizes of 4 ± 4 km (mean ± SD). Plots on the right assume reserve sizes of 2 ± 2 km. The values shown here were used as input for spatially-implicit Keppel island (K) scenarios presented in Figs 1 and 2. Sharp increases in the exchange of adults from fished areas to reserves at high levels of reserve coverage (blue dashed lines) reflected that fewer fish will be exposed to fishing even if the center of their home range is located in a fished area. Sharp declines in exchange at the highest reserve coverages in B and D result from the reserve design procedure, which forcedly switched from being able to represent multiple separate reserves to having either two or a single large reserve. (PNG) [file pbio.2000537.s004.png]

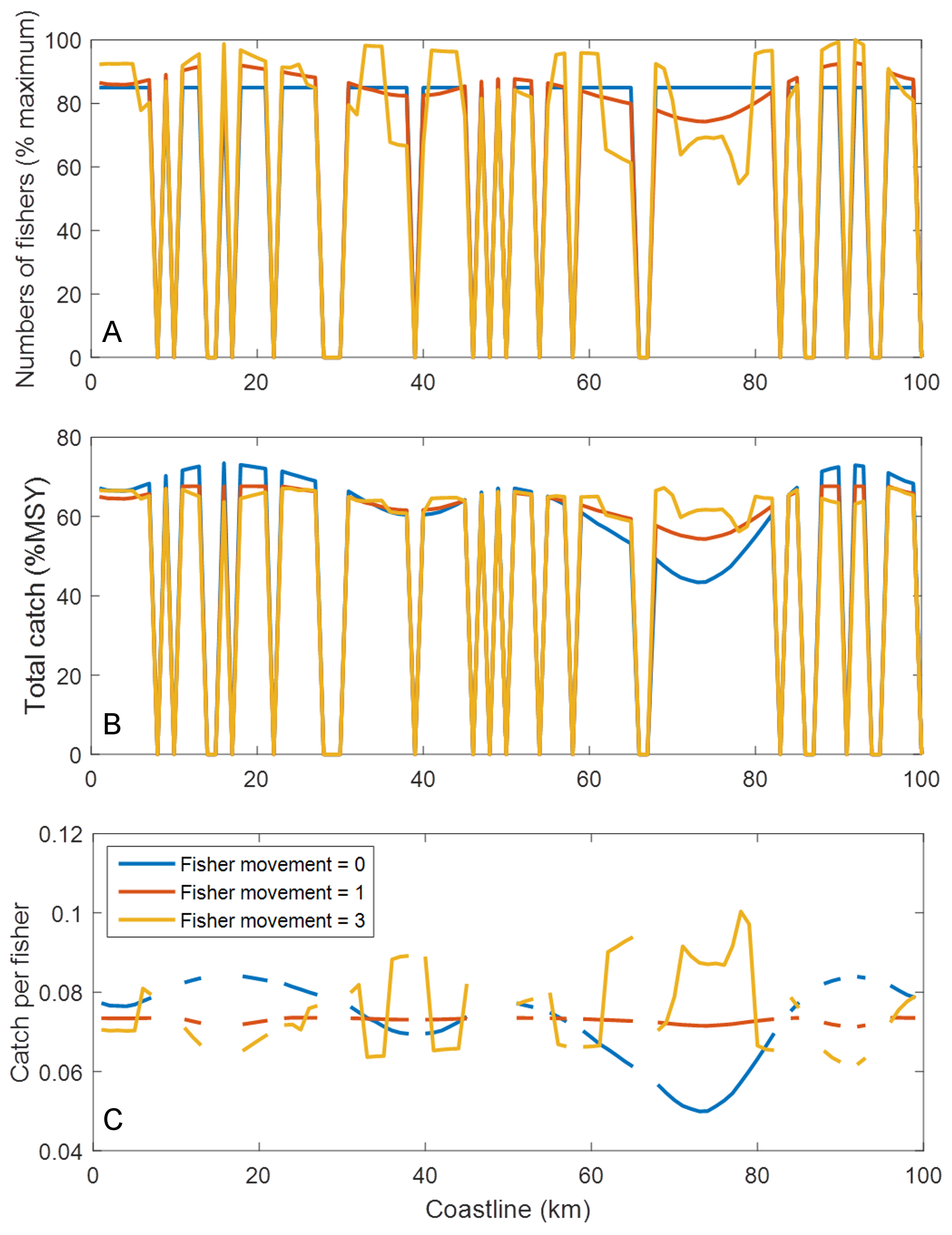

Supplement: S5 Fig — Results are based on our spatially-explicit model for the snapper (Lutjanus carponotatus, see S3 Table for parameterization) and mean reserve sizes of 1-2 km, presenting a snapshot of equilibrium fishery conditions after the enforcement of reserves that cover 25% of an overfished fishing ground (1.5 × FMSY). Setting the fisher movement parameter to 0 resulted in stationary fishing activities and unevenly distributed catch increases per fisher. A fisher movement parameter of 1 resembled an ideal free fisher distribution (catch per fisher is spatially uniform), while a higher value of 3 caused fisher concentrations along the edges of reserves that reversed the distribution of inequality in catch per fisher observed under stationary fishing. (PNG) [file pbio.2000537.s005.png]

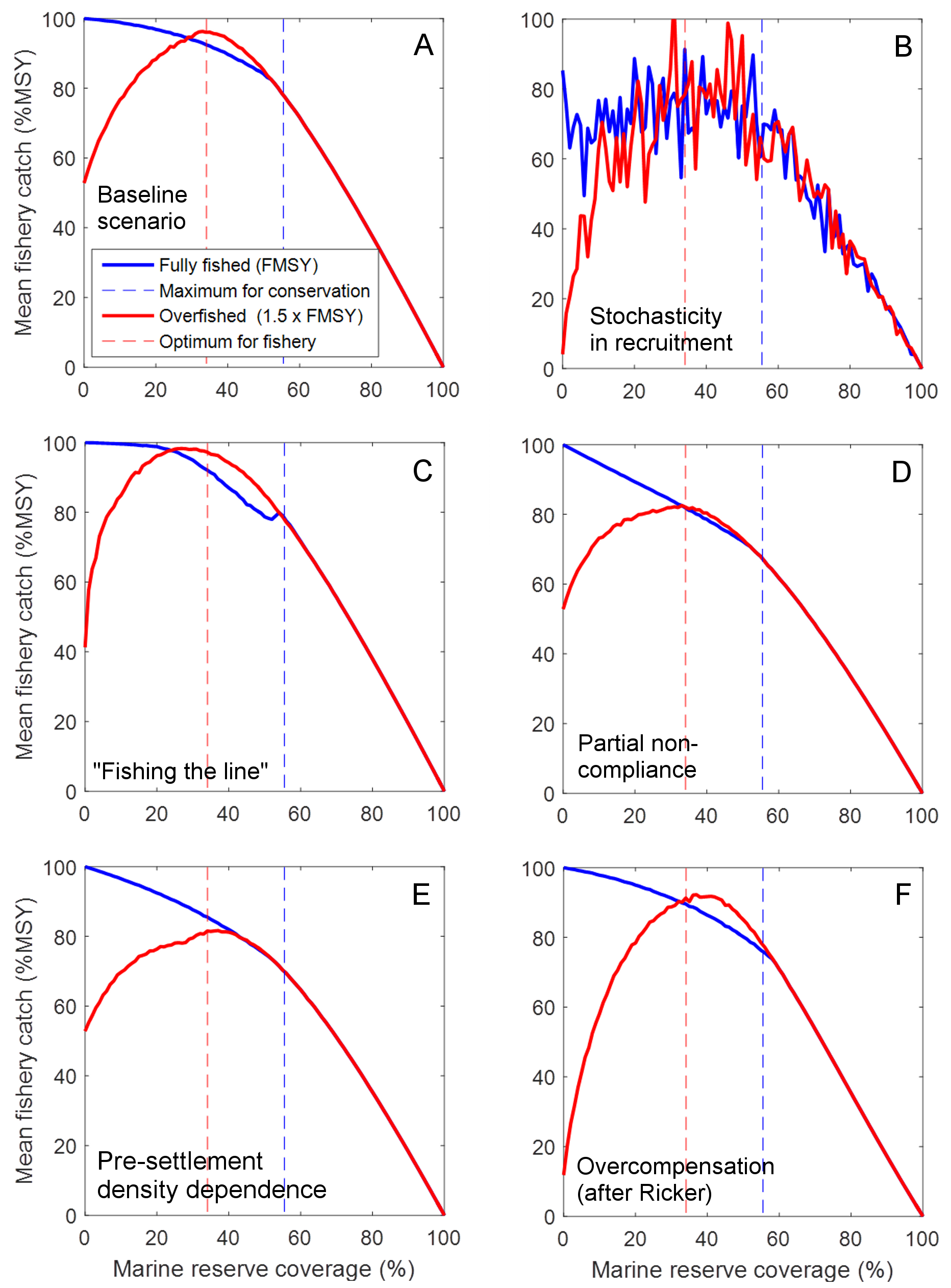

Supplement: S6 Fig — Results show declines in catch under optimal exploitation (blue), and increases in catch under over-exploitation (red) as predicted by our spatially-explicit model in 1 km resolution. All scenarios represent the Spanish flag snapper (Lutjanus carponotatus) and mean reserve sizes of 1–2 km. The baseline scenario (A) is otherwise based on the default parameterization given in S3 Table. In (B) stochasticity in annual recruitment levels is incorporated (0–100%). In (C) fishers concentrate along the edges of reserves (fisher movement parameter = 3). In (D) illegal poaching reduces the recovery potential in reserves by 50%. In (E) density-dependent mortality prior to and after the settlement of larvae is equally important. In (F) Ricker’s recruitment function is used to incorporate minor overcompensation (i.e. reduced recruitment when fish biomass approaches unfished levels). See S1 Table and Materials and Methods for details. (PNG) [file pbio.2000537.s006.png]

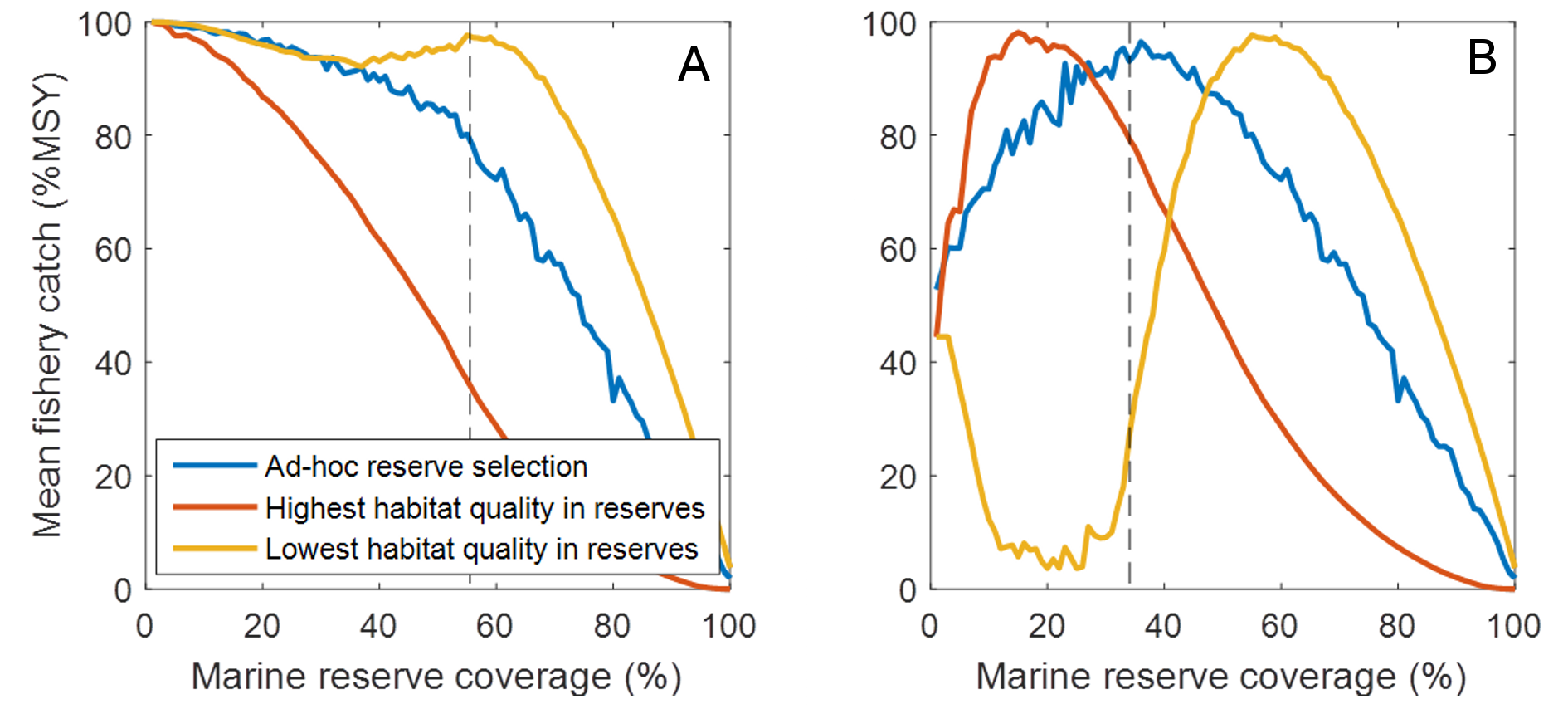

Supplement: S7 Fig — Results refer to the maximum reserve coverage without fisheries costs (A) and the optimum reserve coverage for fishery rebuilding (B) under different reserve siting strategies. The dashed line in A indicates the maximum coverage for our baseline scenario under initially optimal exploitation (Lutjanus carponotatus, see S3 Table). In B, the same conditions are shown but assuming considerable over-exploitation, and with the dashed line referencing the optimum reserve coverage for rebuilding. Ad-hoc (random) reserve siting was found to be unlikely to change management targets, but systematic reserve placement in regions of either lowest or highest habitat quality would. (PNG) [file pbio.2000537.s007.png]

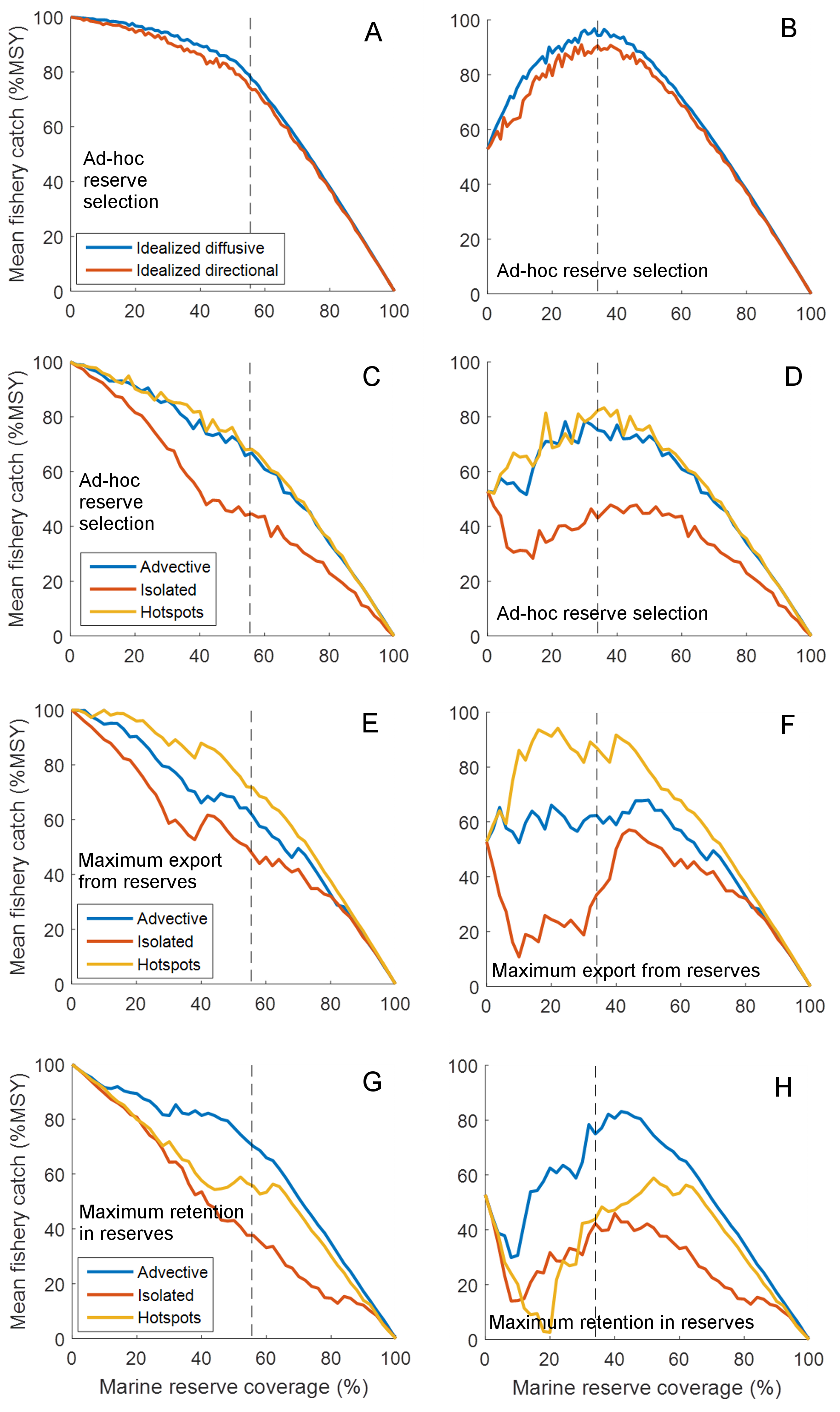

Supplement: S8 Fig — Results refer to the maximum reserve coverage without fisheries costs (left) and the optimum reserve coverage for fisheries rebuilding (right) under different reserve siting strategies. Dashed lines in plots reference reserve coverage targets for our baseline scenario (Lutjanus carponotatus, see S3 Table). In A and B, outcomes for the baseline scenario are compared to a scenario that assumes the same dispersal distances but that dispersal is unidirectional. In C-H, three examples of asymmetric connectivity derived from biophysical simulations of larval dispersal by ocean currents were used. Asymmetric connectivity tends to reduce catch, but not necessarily coverage targets (see D), under ad-hoc reserve site selections. Systematic reserve siting can improve fishery outcomes (E-H). See Materials and Methods for details. (PNG) [file pbio.2000537.s008.png]

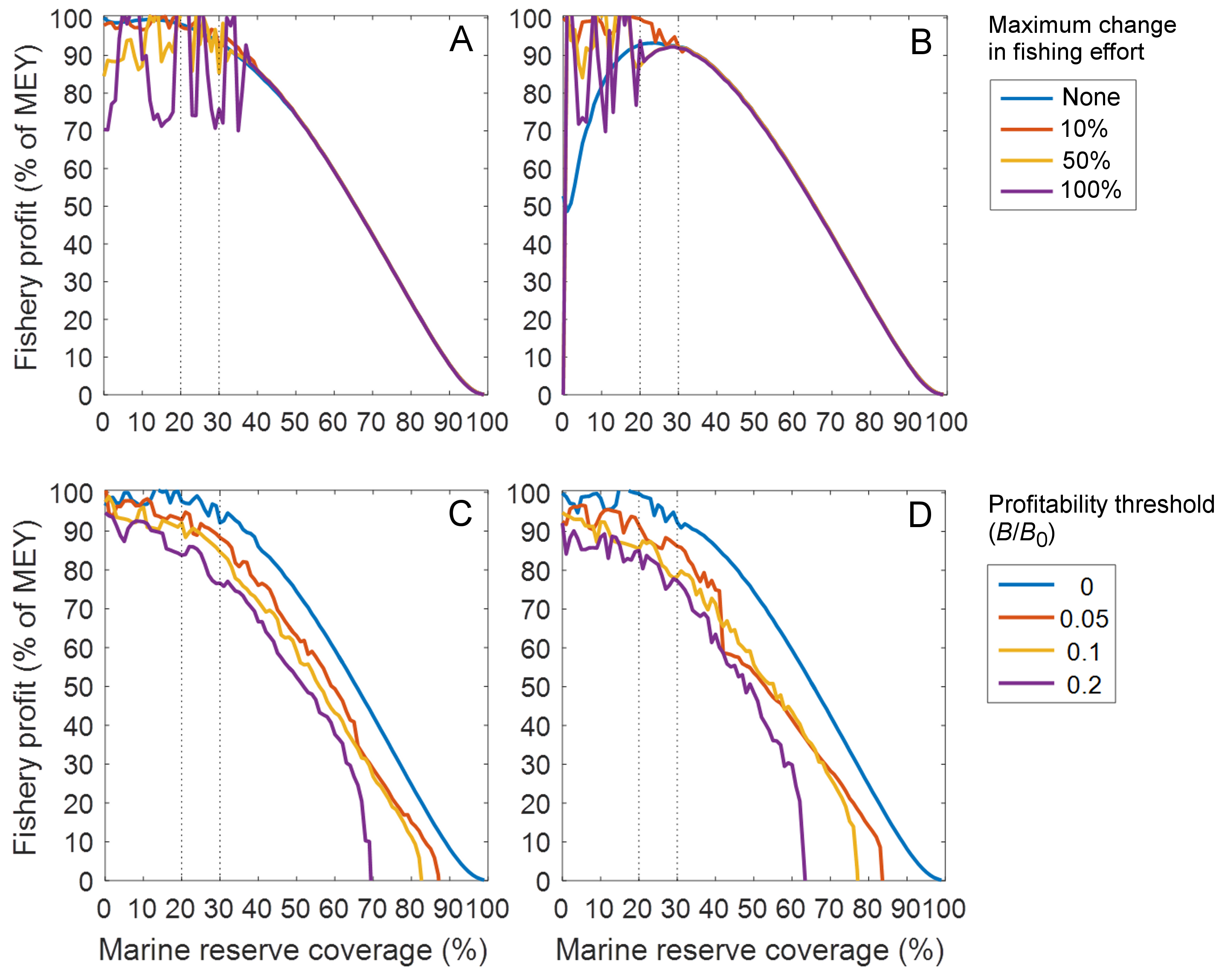

Supplement: S9 Fig — Upper plots assume increasing levels of change in fishing effort relative to current profit under initially optimal exploitation (A) and overexploitation (B). Maximum biannual change in effort ≥50% resulted in fishery collapse if no reserves were implemented. Lower plots (C) and (D) assume maximum changes in fishing effort of 10% (red in A and B) for increasing levels of fishing costs represented by the profitability threshold or “stock effect”. Again, plots to the left and right assume initially optimal exploitation (C) and overexploitation (D). All results are fishery profits 100 years after reserve enforcement. The dashed vertical lines reference our generic biophysical reserve coverage target. MEY, Maximum Economic Yield. See Materials and Methods for details. (PNG) [file pbio.2000537.s009.png]

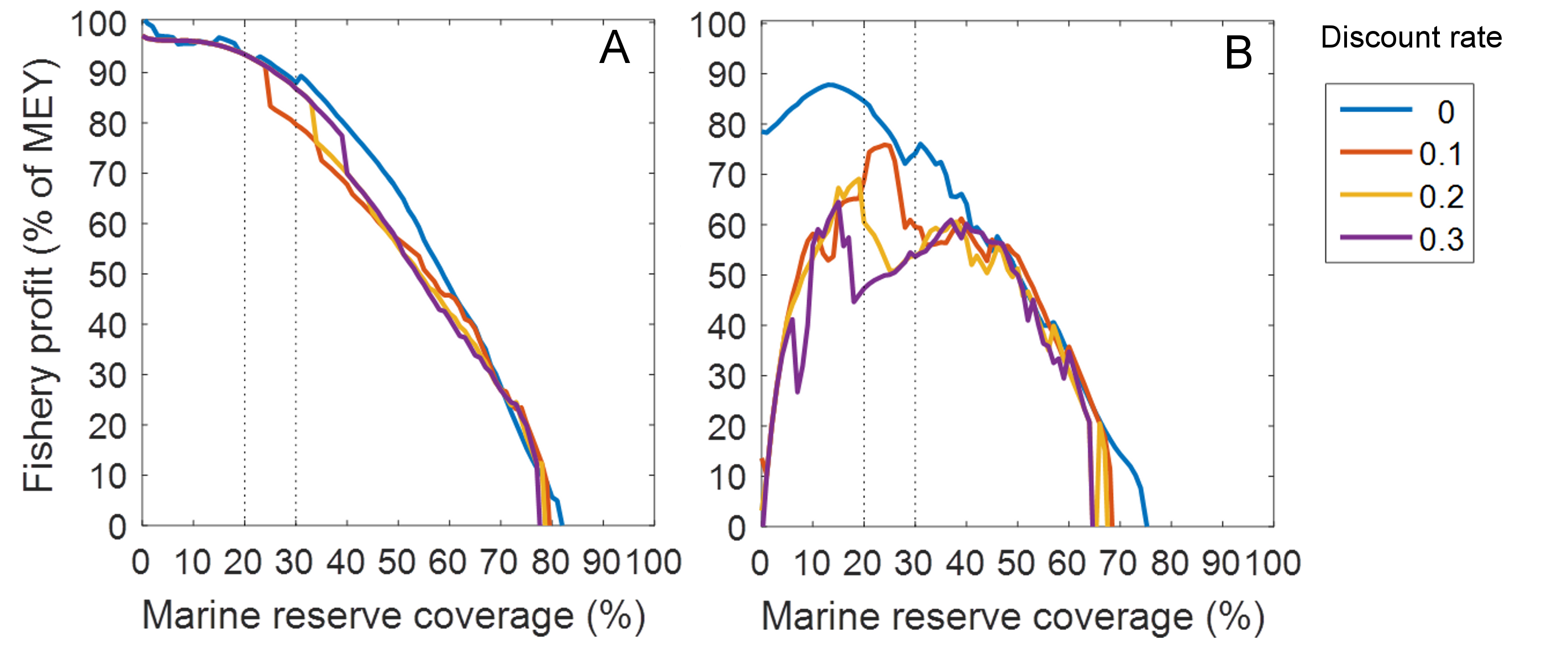

Supplement: S10 Fig — Results are based on the biological assumptions specified for the snapper Lutjanus carponotatus in S3 Table, showing an initially healthy (A) and overfished (B) fishery. Simulations assume (1) dynamic changes in effort (10% maximum biannual change), (2) a profitability threshold (5% of virgin biomass), and (3) variable discount rates. The outcomes are fishery profits 10 years after reserve enforcement. The dashed vertical lines reference our generic biophysical reserve coverage target. MEY, Maximum Economic Yield. See Materials and Methods for details. (PNG) [file pbio.2000537.s010.png]
