# Supplementary material for: Marine Reserve Targets to Sustain and Rebuild Unregulated Fisheries
Source: PLoS Biol. 2017 Jan 5;15(1):e2000537. doi: 10.1371/journal.pbio.2000537 (PMC5215937; doi:10.1371/journal.pbio.2000537)
Supplement: S1 Table — This table is an annotated version of Table 1, providing a description of mechanisms behind simulated fishery trends, as well as additional information on parameter impacts and uncertainties. (DOCX) [file pbio.2000537.s011.docx]

|  |  |  |  | Maximum coverage without fisheries costs | Optimum coverage for fisheries rebuilding | Impact/ Uncertainty | Tested/ Incorporated | Comments | Previous studies |
| --- | --- | --- | --- | --- | --- | --- | --- | --- | --- |
| Species | |  |  |  |  |  |  |  |  |
|  | Natural adult mortality | | | **↑** | **↑** | **++/++** | Yes/Yes | Populations with higher rates of natural adult mortality require higher levels of recruitment to persist. Associated fisheries are therefore more susceptible to catch declines caused by a concentration of fishing effort. However, associated fisheries will also be more susceptible to catch increases through larval dispersal. Measured exchange of larvae across reserve boundaries [[1](#_ENREF_1),[2](#_ENREF_2)] allow to predict that catch increases should outweigh catch declines, causing moderate increases in both maximum reserve coverages without fisheries costs and optimum reserve coverages for fishery rebuilding. Empirical data on natural adult mortality are not widely available, but estimates can be made based on growth and ambient water temperature (e.g. [[3](#_ENREF_3)]). | [[4](#_ENREF_4),[5](#_ENREF_5),[6](#_ENREF_6)] |
|  | Growth | |  | **↓** | **↓** | **+/+** | Yes/Yes | Higher growth rates have implications opposite to those described above for higher mortality rates. That is, fisheries are less susceptible to catch declines caused by the concentration of fishing effort, which, in turn, means that they will also benefit less from catch increases through larval dispersal. Compared to other parameters, associated impacts on reserve coverages are minor. Empirical estimates of growth rates are available for many fishery species and positively correlated with rates of natural adult mortality. | [[4](#_ENREF_4),[5](#_ENREF_5),[6](#_ENREF_6)] |
|  | **Movements** | |  |  |  |  |  |  |  |
|  |  | Larval dispersal | | **↑** | **↓↑** | **+++/++** | Yes/Yes | Larval dispersal benefits the rebuilding of depleted fisheries. It also decreases the susceptibility of healthy fisheries to potential catch declines caused by a concentration of fishing effort. However, reserve coverages for optimal fishery rebuilding might peak at low levels, such as 25%, if target species are maximally productive when their spawning biomass is low [[7](#_ENREF_7)]. The first measurements of realized larval dispersal (i.e. successful settlement of larvae) from reserves to fished areas have recently become available [[1](#_ENREF_1),[2](#_ENREF_2),[8](#_ENREF_8)]. | [[6](#_ENREF_6),[9](#_ENREF_9),[10](#_ENREF_10),[11](#_ENREF_11)] |
|  |  | Juvenile spillover | | **↑** | **↓↑** | **+++/++** | Implicit/  Implicit | The implications of active movements of juveniles from reserves to fished areas are functionally equivalent to the effects described above for larval dispersal, provided that juveniles are not fished [[10](#_ENREF_10)]. If juveniles are targeted by fisheries, their spillover is functionally equivalent to that of adults (see below). Little empirical information on juvenile movements across reserve boundaries is currently available. | [[4](#_ENREF_4),[10](#_ENREF_10)] |
|  |  | Adult spillover | | **↑** | **↑** | **+++/++** | Yes/Yes | Spillover of adults from reserves to fished areas decreases the susceptibility of fisheries to potential catch declines through concentrated fishing effort, but it may require unrealistic levels of coverage for fishery rebuilding. The lowest fishery value of reserves must be expected if fished species have highly mobile adults, but are sensitive to further declines in spawning biomass caused by a concentration of fishing effort outside of reserves. Data on the movements of adult fishes are increasingly available (e.g. [[8](#_ENREF_8)]), but substantial uncertainty remains (see [[12](#_ENREF_12)]). | [[4](#_ENREF_4),[13](#_ENREF_13),[14](#_ENREF_14)] |

|  | **Density-dependence** | |  |  |  |  |  |  |
| --- | --- | --- | --- | --- | --- | --- | --- | --- |
|  |  | Pre-settlement | **↓** | **↑** | **+++/+++** | Yes  (Fig. S5)/No | Fewer larvae will reach and satisfy recruitment capacities at local fishing grounds if density-dependent mortality operates prior to settlement. If pre-settlement density-dependence is substantial, it will therefore decrease the efficency of reserves to compensate for a concentration of fishing effort, resulting in lower catch and maximum reserve coverages for biodiversity conservation. Under strong sensitivity to local larval supply, the same effect will also reduce the capacity of reserves to rebuild fisheries. However, if fished populations are largely insensitive to local larval supply, optimum reserve coverages for fishery rebuilding can increase. There is very little empirical data available to parameterize the magnitude of pre-settlement density-dependence. Importantly, realized dispersal distances of larvae incorporated into our models [[1](#_ENREF_1)] are independent of density-dependence effects prior to settlement. | [[6](#_ENREF_6),[15](#_ENREF_15)] |
|  |  | Post-settlement |  |  |  |  |  |  |
|  |  | Intra-cohort | **↓** | **↓** | **+++/++** | Yes/Yes | It is widely acknowledged that density-dependent mortality after settlement is substantial [[16](#_ENREF_16)], which implies that healthy fish populations are not sensitive to larval supply. However, under full exploitation or over-exploitation, catch might decline or increase sharply for a given change in larval supply, resulting in the overall greatest sensitivity of fisheries to reserve enforcement. Fish populations that are more sensitive to larval supply when they are healthy experience greater declines for the same absolute level of fishing pressure. However, their fisheries are relatively less sensitive to changes in larval supply (see Fig. S1) and, thus, reserve enforcement. Several meta-analyses allow to parameterize Beverton-Holt and Ricker functions to represent post-settlement density dependence in models (e.g. [[17](#_ENREF_17),[18](#_ENREF_18)]), but high uncertainty remains. | [[6](#_ENREF_6),[15](#_ENREF_15),[19](#_ENREF_19),[20](#_ENREF_20)] |
|  |  | Inter-cohort | **↓** | **↑** | **+++/+++** | Yes  (Fig. S5)/No | Inter-cohort density-dependent mortality after settlement refers to trophic and behavioural interactions among different age groups. Cannibalism of adults on larvae or juveniles is a common example, resulting in an effect known as "overcompensation". Overcompensation means that fewer recruits will enter adult populations while their biomass approaches unfished levels, which is generally represented in models based on Ricker's recruitment function. Intuitively, strong overcompensation can lead to substantial increases in the level of reserve coverage required to rebuild biomass and catch. Strong overcompensation will also tend to reduce maximum reserve coverages without fisheries costs, because it implies that deviations from optimum yields will be steep. However, empirical support for strong overcompensation is limited, with empirical fits of the Ricker function generally resembling a Beverton-Holt stock-recruitment relationship (e.g. [[18](#_ENREF_18)]). | [[6](#_ENREF_6),[21](#_ENREF_21),[22](#_ENREF_22),[23](#_ENREF_23)] |
|  |  | Inter-specific | **↓↑** | **↓↑** | **+++/+++** | No/No | Density dependent mortality caused by interactions among different species can have the same implications as described above for both intra- and inter-cohort density-dependence. The effect of such interactions could be even more important than intra-specific interactions. However, there is very limited empirical data to support the parameterization of inter-specific density-dependence, both in terms of magnitude and direction, given that a disadvantage for one species would likely result in a benefit to another. | [[24](#_ENREF_24)] |
|  | Fishery | |  |  |  |  |  |  |
|  |  | **Exploitation level** | **↓** | **↑** | **+++/++** | Yes/Yes | With no catch regulations in place, the concentration of fishing effort under increasing coverage of reserves is likely to cause under-exploited fisheries to approach the maximum sustainable yield. Under full exploitation or moderate over-exploitation, reserve enforcement is likely to reduce catch unless fish movements from reserves into fished areas can compensate for the concentration of fishing effort outside of reserves. Under heavy overfishing, reserve enforcement is likely to increase catch up to a point when larval dispersal and spillover from reserves convey no further catch increases relative to the confounding effect of further concentration of fishing effort. | [[4](#_ENREF_4),[5](#_ENREF_5),[20](#_ENREF_20)] |
|  |  | Effort displacement | **↑** | **↑** | **+++/++** | Yes/Yes | Perhaps counterintuitively, the displacement of fishing effort from reserves to fished areas enables higher reserve coverages without impacts on healthy fisheries. It also requires higher reserve coverages to rebuild depleted fisheries. If the fishing effort in formerly unprotected locations would simply disappear, fisheries targeting initially healthy populations will be overall less productive. If fisheries are depleted prior to reserve enforcement, the maximum sustainable yield will be achieved at lower reserve coverages, because catch increases delivered by growing spawning biomass and larval dispersal from reserves do not have to compensate for the negative impacts of fisher concentration. | [[4](#_ENREF_4),[25](#_ENREF_25)]; |
|  |  | Fisher mobility | **↑** | **↓** | **++/++** | Yes  (Fig. S4)/No | The capacity of fishers to harvest their target species optimally (ideal free distribution) increases the magnitude of catch increases that reserves can provide. Maximum catch increases can also be achieved at lower levels of reserve coverage if fishers effectively exploit productivity rather than if they are stationary. Similarly, potential catch declines of healthy fisheries tend to be reduced and might occur at higher levels of coverage if fishers are non-stationary. A common assumption is that fishers perceive optimal yields around marine reserves, thus preferentially exploiting the edges of reserves ("fishing the line" [[26](#_ENREF_26)]). However, fisher behaviour remains difficult to generalize, specifically if conditions are complex and fisher movements are not "free" [[27](#_ENREF_27)]. | [[10](#_ENREF_10),[13](#_ENREF_13),[28](#_ENREF_28),[29](#_ENREF_29)] |
|  |  | Partial non-compliance | ? | ↑ | ?/++ | Yes  (Fig. S5)/No | Partial non-compliance requires a potentially substantial increase in the level of reserve coverage required to rebuild fisheries optimally. The implications of partial non-compliance for levels of reserve coverage without any costs to healthy fisheries are less clear, because the relationship between levels of poaching and harmful fisher concentrations outside of reserves is likely to be negative. While partial non-compliance is highly likely, there are generally no quantitative estimates of poaching, specifically not in complex situations where it is most likely to occur. We believe that the management target should be to try and eliminate any poaching. | [[30](#_ENREF_30)] |
|  |  | **Catch regulations** | ↑ | ↓ | +++/+ | No/No | Fishery regulations other than reserves should increase the level of reserve coverage that healthy fisheries can tolerate, because negative impacts caused by the concentration of effort in fished areas will be mitigated or completely avoided. Similarly, substantially lower levels of reserve coverage can be required to rebuild depleted fisheries if other regulations function to protect adult spawning biomass and ecosystems from fishing. | [[13](#_ENREF_13),[20](#_ENREF_20)] |
|  |  | Socio-economic context | ↓↑ | ↓↑ | ++/+++ | Yes  (Fig. S9-S10)/  Implicit | Our study focused on the situation of fishers with a high dependence on fishing and no or very limited alternative livelihood opportunities. If fisher livelihoods are more diverse or flexible, fishers may enter and exit fishing activities myopically in response to current profits [[29](#_ENREF_29),[31](#_ENREF_31)]. Moderate variation in fishing effort under open access conditions can stabilize fishery profits around long-term optima, while highly dynamic responses of fishers might result in fishery collapse (the “tragedy of the commons“ scenario) [[32](#_ENREF_32)]. Generic reserve coverage targets of 20-30% can help ensure that long-term profits close to the maximum economic yield are sustained in the long-term, regardless of initial fishing intensity and whether fishing effort is constant or highly dynamic (Fig. S9A-B). Given that fishing costs are likely to increase as the biomass of fished populations declines, thresholds in profitability (the so-called “stock effect”) [[22](#_ENREF_22)] might reduce maximum reserve coverage targets for biodiversity conservation benefits without fishery costs (Fig. 9C-D). In contrast, optimum reserve coverage targets for fishery rebuilding remain unchanged, but fisheries might not always become profitable again. However, generalizing such implications of socio-economic context is challenging as suitable reserve coverage targets will depend on interactions between multiple factors, including (1) local costs and revenues for a given catch and fishery status, (2) the change in effort (access) for a given change in profit, (3) the time over which fisheries are assessed, and (4) the change in costs and revenue over this time (see e.g. Fig. S10). | [[11](#_ENREF_11),[22](#_ENREF_22),[29](#_ENREF_29),[33](#_ENREF_33),[34](#_ENREF_34)] |
|  | Environment | |  |  |  |  |  |  |
|  |  | Stochasticity in recruitment | **↑** | **↑** | **++/++** | Yes  (Fig. S5)/No | Fluctuations in recruitment caused by climatic and other environmental variations appear to be likely and strong [[18](#_ENREF_18),[35](#_ENREF_35),[36](#_ENREF_36)]. Fish biomass protected in reserves could mitigate such fluctuations by ensuring higher minimum levels of larval supply [[5](#_ENREF_5)]. Achieving such a buffering function of reserves will require higher reserve coverages. However, the magnitude and causes of recruitment variation are still highly uncertain. Moreover, it is not clear to what extent a relative increase in protected biomass would help stabilize and sustain the long-term productivity of fishery catch. | [[5](#_ENREF_5),[37](#_ENREF_37)] |
|  |  | Gradients in habitat quality | **↓↑** | **↓↑** | **++/+++** | Yes  (Fig. S6)/No | Gradients in habitat quality are likely to be encountered whenever reserve are implemented, but such gradients do not change the validity of generic advice on reserve coverage targets as long as the placement of reserves is non-systematic, i.e. covering areas of both low and high habitat quality in similar frequency. However, reserves themselves could cause or intensify habitat quality gradients, because the concentration of fishing effort degrades habitat quality in fished areas. In this case, fewer than expected larvae exported from reserves might recruit into adjacent fisheries. Another possible effect is that an initial increase in habitat quality, fish biomass and larval export within reserve boundaries is followed by an increase in adult spillover from reserves (see below under behavioural interactions). | [[10](#_ENREF_10),[38](#_ENREF_38),[39](#_ENREF_39)] |
|  |  | Asymmetric connectivity | **↓** | ↓ | **++/+++** | Yes  (Fig. S7)/No | Asymmetry in the connectivity of fish populations caused by the dynamic dispersal of fish larvae with ocean currents tends to increase the sensitivity of fisheries productivity to reserve enforcement. That is, systematic reserve network designs could fundamentally impact fishery outcomes for good or bad. A forcedly poorly informed placement of reserves with respect to asymmetric connectivity (likely in many regions) could cause catch declines below PGY under lower reserve coverages if fisheries are healthy. However, there would be little or no impact on reserve coverage targets to rebuild depleted fisheries. The first empirical larval dispersal matrices are yet to be published, leaving researchers and managers largely unable to incorporate this critical process into management advice. | [[33](#_ENREF_33),[40](#_ENREF_40)] |
|  |  | **Trophic interactions** | **↓↑** | **↓↑** | **+++/++** | No/No | Trophic cascades in and adjacent to reserves can benefit some and disadvantage other species, making generic predictions under complex fishery conditions difficult or impossible. However, under heavy overfishing, both predator and prey species are likely to increase in abundance [[41](#_ENREF_41)]. Moreover, the vast majority of species across taxonomic and functional groups has been found to increase in both density and biomass within reserves [[42](#_ENREF_42),[43](#_ENREF_43),[44](#_ENREF_44)]. Presuming that local reserves sizes enable some protection of adults but still allow for larval export, complex, multi-species fisheries should thus experience net benefits. | [[28](#_ENREF_28),[41](#_ENREF_41),[45](#_ENREF_45)] |
|  |  | Behavourial interactions | **↓↑** | **↓↑** | **?/+++** | No/No | Behavioural interactions among individuals of the same and other species could have similar implications for the fishery functioning of reserves as trophic interactions. However, the impact of such interactions is largely unknown. One potentially important cause of density-dependent movements of adults from reserves to fished areas is an increase in competition while fish densities approach unfished levels [[46](#_ENREF_46)]. |  |
|  | Reserve network design | |  |  |  |  |  |  |
|  |  | **Location of reserves** | **↓↑** | **↓↑** | **+++/+++** | Yes  (Fig. S6-7)/  No | Spatial prioritization of reserve locations, for example, based on dispersal connectivity or habitat quality, can fundamentally impact fisheries productivity. This includes the level of reserve coverages with no costs to healthy fisheries as well as the optimum reserve coverage required for maximum catch increases from depleted fisheries. If the prioritization process was entirely fishery-focused and environmental information was robust, maximum yields are likely to be achieved by closing overall lower proportions of fishing grounds. Similarly, under such conditions, a focus on biodiversity conservation might enable covering a very high proportion of fishing grounds in reserves without harming local fisher livelihoods or economic returns. | [[33](#_ENREF_33),[40](#_ENREF_40)] |
|  |  | **Size of reserves** | **↓** | **↓** | **+++/+** | Yes/Yes | Increasing the size of individual reserves will reduce their capacity to subsidize fishery catch through larval dispersal or adult spillover. However, reserves need to be large enough to protect some adults for any conservation and fishery benefits to accrue. Decisions on reserve size and reserve coverage are thus linked in terms of fishery impact, but they are physically independent unless fishing grounds are small or reserve coverage is high (>50%). | [[9](#_ENREF_9),[47](#_ENREF_47)] |

# References

1. Harrison HB, Williamson DH, Evans RD, Almany GR, Thorrold SR, et al. (2012) Larval export from marine reserves and the recruitment benefit for fish and fisheries. Curr Biol 22: 1023-1028.

2. Jones GP (2015) Mission impossible: unlocking the secrets of coral reef fish dispersal. In: Mora C, editor. Ecology of fishes on coral reefs. Cambridge, UK: Cambridge University Press. pp. 16-27.

3. Pauly D (1980) On the interrelationships between natural mortality, growth parameters, and mean environmental temperature in 175 fish stocks. ICES J Mar Sci 39: 175-192.

4. Beverton RJH, Holt SJ (1957) On the dynamics of exploited fish populations. Fishery Investigations Series 2: Sea Fisheries 19.

5. Mangel M (2000) On the fraction of habitat allocated to marine reserves. Ecol Lett 3: 15-22.

6. Gaylord B, Gaines SD, Siegel DA, Carr MH (2005) Marine reserves exploit population structure and life history in potentially improving fisheries yields. Ecol Appl 15: 2180-2191.

7. Hilborn R (2010) Pretty good yield and exploited fishes. Mar Policy 34: 193-196.

8. Green AL, Maypa AP, Almany GR, Rhodes KL, Weeks R, et al. (2015) Larval dispersal and movement patterns of coral reef fishes, and implications for marine reserve network design. Biol Rev Camb Philos Soc 90: 1215-1247.

9. Hastings A, Botsford LW (2003) Comparing designs of marine reserves for fisheries and for biodiversity. Ecol Appl 13: S65-S70.

10. Walters CJ, Hilborn R, Parrish R (2007) An equilibrium model for predicting the efficacy of marine protected areas in coastal environments. Can J Fish Aquat Sci 64: 1009-1018.

11. Sanchirico JN, Malvadkar U, Hastings A, Wilen JE (2006) When are no-take zones an economically optimal fishery management strategy? Ecol Appl 16: 1643-1659.

12. Sale PF, Cowen RK, Danilowicz BS, Jones GP, Kritzer JP, et al. (2005) Critical science gaps impede use of no-take fishery reserves. Trends Ecol Evol 20: 74-80.

13. Hilborn R, Micheli F, De Leo GA (2006) Integrating marine protected areas with catch regulation. Can J Fish Aquat Sci 63: 642-649.

14. Moffitt EA, Botsford LW, Kaplan DM, O'Farrell MR (2009) Marine reserve networks for species that move within a home range. Ecol Appl 19: 1835-1847.

15. McGilliard CR, Hilborn R (2008) Modeling no-take marine reserves in regulated fisheries: assessing the role of larval dispersal. Can J Fish Aquat Sci 65: 2509-2523.

16. Hixon MA, Webster MS (2002) Density dependence in reef fish populations. In: Sale PF, editor. Coral reef fishes: dynamics and diversity in a complex ecosystem. San Diego, California, USA: Academic Press. pp. 303-325.

17. Myers RA, Bowen KG, Barrowman NJ (1999) Maximum reproductive rate of fish at low population sizes. Can J Fish Aquat Sci 56: 2404-2419.

18. Cury PM, Fromentin J-M, Figuet S, Bonhommeau S (2014) Resolving Hjort's Dilemma How Is Recruitment Related to Spawning Stock Biomass in Marins Fish? Oceanography 27: 42-47.

19. Hastings A, Botsford LW (1999) Equivalence in yield from marine reserves and traditional fisheries management. Science 284: 1537-1538.

20. Hart DR (2006) When do marine reserves increase fishery yield? Can J Fish Aquat Sci 63: 1445-1449.

21. White C, Kendall BE (2007) A reassessment of equivalence in yield from marine reserves and traditional fisheries managament. Oikos 116: 2039-2043.

22. White C, Kendall BE, Gaines S, Siegel DA, Costello C (2008) Marine reserve effects on fishery profit. Ecol Lett 11: 370-379.

23. Hart DR, Sissenwine MP (2009) Marine reserve effects on fishery profits: a comment on White et al. (2008). Ecol Lett 12: E9-E11.

24. Baskett ML, Yoklavich M, Love MS (2006) Predation, competition, and the recovery of overexploited fish stocks in marine reserves. Can J Fish Aquat Sci 63: 1214-1229.

25. Halpern BS, Gaines SD, Warner RR (2004) Confounding effects of the export of production and the displacement of fishing effort from marine reserves. Ecol Appl 14: 1248-1256.

26. Kellner JB, Tetreault I, Gaines SD, Nisbet RM (2007) Fishing the line near marine reserves in single and multispecies fisheries. Ecol Appl 17: 1039-1054.

27. Abernethy KE, Allison EH, Molloy PP, Cote IM (2007) Why do fishers fish where they fish? Using the ideal free distribution to understand the behaviour of artisanal reef fishers. Can J Fish Aquat Sci 64: 1595-1604.

28. Walters C (2000) Impacts of dispersal, ecological interactions, and fishing effort dynamics on efficacy of marine protected areas: how large should protected areas be? Bull Mar Sci 66: 745-757.

29. Sanchirico JN, Wilen JE (2001) A bioeconomic model of marine reserve creation. J Environ Econ Manage 42: 257-276.

30. Le Quesne WJF (2009) Are flawed MPAs any good or just a new way of making old mistakes? ICES Journal of Marine Science: Journal du Conseil 66: 132-136.

31. Gordon HS (1954) Economic theory of a common property resource: the fishery. Journal of Political Economy 75: 124–142.

32. Berkes F (1985) Fishermen and ‘The Tragedy of the Commons’. Environ Conserv 12: 199-206.

33. Rassweiler A, Costello C, Siegel DA (2012) Marine protected areas and the value of spatially optimized fishery management. Proceedings of the National Academy of Sciences 109: 11884-11889.

34. Tuck GN, Possingham HP (1994) Optimal Harvesting Strategies for a Metapopulation. Bull Math Biol 56: 107-127.

35. Vert-pre KA, Amoroso RO, Jensen OP, Hilborn R (2013) Frequency and intensity of productivity regime shifts in marine fish stocks. Proceedings of the National Academy of Sciences 110: 1779-1784.

36. Szuwalski CS, Vert-Pre KA, Punt AE, Branch TA, Hilborn R (2014) Examining common assumptions about recruitment: a meta-analysis of recruitment dynamics for worldwide marine fisheries. Fish Fish: n/a-n/a.

37. West CD, Dytham C, Righton D, Pitchford JW (2009) Preventing overexploitation of migratory fish stocks: the efficacy of marine protected areas in a stochastic environment. ICES J Mar Sci 66: 1919-1930.

38. Roberts CM, Sargant H (2002) Fishery benefits of fully protected marine reserves: why habitat and behavior are important. Nat Resour Model 15: 487-507.

39. Rodwell LD, Barbier EB, Roberts CM, McClanahan TR (2003) The importance of habitat quality for marine reserve fishery linkages. Can J Fish Aquat Sci 60: 171-181.

40. Costello C, Rassweiler A, Siegel D, De Leo G, Micheli F, et al. (2010) The value of spatial information in MPA network design. Proc Natl Acad Sci U S A 107: 18294-18299.

41. Micheli F, Amarasekare P, Bascompte J, Gerber LR (2004) Including species interactions in the design and evaluation of marine reserves: some insights from a predator-prey model. Bull Mar Sci 74: 653-669.

42. Halpern BS (2003) The impact of marine reserves: Do reserves work and does reserve size matter? Ecol Appl 13: S117-S137.

43. Micheli F, Halpern BS, Botsford LW, Warner RR (2004) Trajectories and correlates of community change in no-take marine reserves. Ecol Appl 14: 1709-1723.

44. Lester SE, Halpern BS, Grorud-Colvert K, Lubchenco J, Ruttenberg BI, et al. (2009) Biological effects within no-take marine reserves: a global synthesis. Marine Ecology-Progress Series 384: 33-46.

45. Walters C, Pauly D, Christensen V (1999) Ecospace: prediction of mesoscale spatial patterns in trophic relationships of exploited ecosystems, with emphasis on the impacts of marine protected areas. Ecosystems 2: 539-554.

46. Abesamis RA, Russ GR (2005) Density-dependent spillover from a marine reserve: long-term evidence. Ecol Appl 15: 1798-1812.

47. Edgar GJ, Stuart-Smith RD, Willis TJ, Kininmonth S, Baker SC, et al. (2014) Global conservation outcomes depend on marine protected areas with five key features. Nature 506: 216-220.
