# Supplementary material for: Marine Reserve Targets to Sustain and Rebuild Unregulated Fisheries
Source: PLoS Biol. 2017 Jan 5;15(1):e2000537. doi: 10.1371/journal.pbio.2000537 (PMC5215937; doi:10.1371/journal.pbio.2000537)
Supplement: S2 Table — Values are means ± standard deviations as presented in Fig 1, but showing results for all combinations of fish movement scenarios. Percentages of catch and biomass refer to maxima, i.e. maximum sustainable yield and unfished biomass. Minimum and maximum reserve coverages under variable levels of overfishing intensity are highlighted in bold. (DOCX) [file pbio.2000537.s012.docx]

| Reference point | | | Exchange | | Coverage  (%) | | Fisheries  (%) | | Catch  (%) | Protected biomass (%) | | Unprotected biomass (%) |  |  |
| --- | --- | --- | --- | --- | --- | --- | --- | --- | --- | --- | --- | --- | --- | --- |
| Maximum without fisheries costs under moderate overfishing | | | None | | **10.7 ± 3.77** | | 97.1 | | 81.0 ± 0.71 | 100 | | 12.8 ± 6.19 |  |  |
|  |  |  | A1 | | 18.0 ± 10.9 | | 97.1 | | 80.8 ± 0.69 | 76.2 ± 10.2 | | 11.2 ± 6.04 |  |  |
|  |  |  | A2 | | 28.2 ± 19.1 | | 97.1 | | 80.7 ± 0.61 | 56.0 ± 10.5 | | 9.23 ± 5.27 |  |  |
|  |  |  | A3 | | 36.0 ± 23.3 | | 97.1 | | 80.7 ± 0.57 | 38.4 ± 9.72 | | 7.60 ± 4.35 |  |  |
|  |  |  | L1 | | 16.5 ± 6.04 | | 97.1 | | 80.6 ± 0.46 | 98.2 ± 1.34 | | 11.8 ± 6.15 |  |  |
|  | | | L2 | | 34.0 ± 9.18 | | 97.1 | | 80.3 ± 0.21 | 95.6 ± 2.69 | | 7.73 ± 6.24 |  |  |
|  | | | L3 | | 51.1 ± 8.73 | | 97.1 | | 80.5 ± 0.30 | 92.1 ± 4.02 | | 3.27 ± 4.49 |  |  |
|  | | | A1L1 | | 25.7 ± 14.0 | | 97.1 | | 80.5 ± 0.44 | 77.2 ± 9.93 | | 9.85 ± 5.86 |  |  |
|  | | | A1L2 | | 44.6 ± 14.4 | | 97.1 | | 80.3 ± 0.23 | 80.2 ± 9.12 | | 5.44 ± 5.48 |  |  |
|  | | | A1L3 | | 59.3 ± 9.61 | | 97.1 | | 80.6 ± 0.39 | 81.9 ± 8.15 | | 1.96 ± 3.56 |  |  |
|  | | | A2L1 | | 36.1 ± 21.4 | | 97.1 | | 80.5 ± 0.44 | 60.1 ± 11.6 | | 7.63 ± 4.75 |  |  |
|  | | | A2L2 | | 55.4 ± 19.8 | | 97.1 | | 80.5 ± 0.38 | 68.3 ± 12.8 | | 3.54 ± 3.76 |  |  |
|  | | | A2L3 | | 67.0 ± 10.9 | | 97.1 | | 80.8 ± 0.56 | 72.8 ± 13.0 | | 1.09 ± 2.23 |  |  |
|  | | | A3L1 | | 42.7 ± 26.0 | | 97.1 | | 80.6 ± 0.55 | 45.0 ± 11.6 | | 6.32 ± 3.77 |  |  |
|  | | | A3L2 | | 60.6 ± 25.9 | | 97.1 | | 80.7 ± 0.67 | 57.6 ± 14.3 | | 3.03 ± 2.63 |  |  |
|  | | | A3L3 | | **73.0 ± 13.2** | | 97.1 | | 81.0 ± 0.79 | 65.2 ± 16.2 | | 0.66 ± 1.33 |  |  |
| Optimum for fishery rebuilding under moderate overfishing | | | None | | NA | | NA | | NA | NA | | NA |  |  |
|  |  |  | A1 | | NA | | NA | | NA | NA | | NA |  |  |
|  |  |  | A2 | | NA | | NA | | NA | NA | | NA |  |  |
|  |  |  | A3 | | **55 ± 0.92** | | 0.31 | | 34.2 ± 4.38 | 37.5 ± 13.3 | | 0.00 ± 0.02 |  |  |
|  | | | L1 | | 29.4 ± 2.98 | | 1.51 | | 61.7 ± 8.43 | 99.6 ± 0.14 | | 0.91 ± 1.47 |  |  |
|  | | | L2 | | **8.73 ± 8.22** | | 15.1 | | 91.9 ± 5.01 | 93.8 ± 5.58 | | 9.99 ± 8.49 |  |  |
|  | | | L3 | | 14.4 ± 8.71 | | 90.1 | | 98.8 ± 1.67 | 82.3 ± 8.34 | | 14.9 ± 7.54 |  |  |
|  | | | A1L1 | | 31.4 ± 3.17 | | 1.54 | | 63.0 ± 8.02 | 79.8 ± 11.1 | | 0.84 ± 1.47 |  |  |
|  | | | A1L2 | | 11.0 ± 8.35 | | 19.0 | | 93.8 ± 5.04 | 71.4 ± 11.4 | | 13.6 ± 9.42 |  |  |
|  | | | A1L3 | | 19.4 ± 9.56 | | 91.5 | | 98.8 ± 1.67 | 67.8 ± 9.19 | | 13.9 ± 7.76 |  |  |
|  | | | A2L1 | | 31.1 ± 9.01 | | 1.96 | | 70.5 ± 13.9 | 56.8 ± 14.2 | | 9.14 ± 10.5 |  |  |
|  | | | A2L2 | | 20.8 ± 10.6 | | 17.1 | | 94.2 ± 5.36 | 55.7 ± 12.0 | | 14.4 ± 9.05 |  |  |
|  | | | A2L3 | | 29.6 ± 12.3 | | 90.2 | | 98.8 ± 1.69 | 56.3 ± 11.3 | | 11.1 ± 7.93 |  |  |
|  | | | A3L1 | | 39.2 ± 5.67 | | 1.86 | | 68.5 ± 8.14 | 42.2 ± 16.0 | | 3.78 ± 7.83 |  |  |
|  | | | A3L2 | | 35.1 ± 12.3 | | 8.86 | | 91.1 ± 5.46 | 45.5 ± 14.3 | | 7.65 ± 8.31 |  |  |
|  | | | A3L3 | | 49.3 ± 17.1 | | 80.2 | | 98.8 ± 1.62 | 49.9 ± 14.7 | | 5.30 ± 6.30 |  |  |
| Maximum without fisheries costs under considerable overfishing | None | | | **5.14 ± 2.95** | | 48.4 | | 80.9 ± 0.61 | | | 100 | 7.66 ± 6.00 | |  |
|  | A1 | | | 12.4 ± 10.5 | | 48.4 | | 80.5 ± 0.43 | | | 71.3 ± 10.9 | 6.57 ± 5.55 | |  |
|  | A2 | | | 25.5 ± 20.8 | | 48.4 | | 80.3 ± 0.31 | | | 48.4 ± 10.9 | 4.97 ± 4.62 | |  |
|  | A3 | | | 31.0 ± 23.4 | | 48.4 | | 80.3 ± 0.29 | | | 26.8 ± 11.2 | 4.43 ± 4.07 | |  |
|  | L1 | | | 10.3 ± 5.90 | | 48.4 | | 80.4 ± 0.33 | | | 97.9 ± 1.67 | 7.34 ± 5.75 | |  |
|  | L2 | | | 34.3 ± 9.74 | | 48.4 | | 80.3 ± 0.18 | | | 95.3 ± 2.97 | 4.14 ± 4.95 | |  |
|  | L3 | | | 53.3 ± 8.44 | | 48.4 | | 80.5 ± 0.31 | | | 92.0 ± 4.11 | 1.47 ± 2.86 | |  |
|  | A1L1 | | | 23.3 ± 16.0 | | 48.4 | | 80.3 ± 0.23 | | | 73.8 ± 10.5 | 5.45 ± 5.10 | |  |
|  | A1L2 | | | 50.5 ± 14.1 | | 48.4 | | 80.3 ± 0.24 | | | 81.3 ± 9.39 | 2.08 ± 3.62 | |  |
|  | A1L3 | | | 64.4 ± 8.49 | | 48.4 | | 80.7 ± 0.44 | | | 83.3 ± 8.10 | 0.53 ± 1.65 | |  |
|  | A2L1 | | | 37.3 ± 24.4 | | 48.4 | | 80.3 ± 0.25 | | | 55.7 ± 13.3 | 3.58 ± 3.84 | |  |
|  | A2L2 | | | 64.2 ± 18.3 | | 48.4 | | 80.5 ± 0.47 | | | 72.2 ± 13.6 | 0.98 ± 2.09 | |  |
|  | A2L3 | | | 73.7 ± 8.08 | | 48.4 | | 80.9 ± 0.64 | | | 76.7 ± 12.4 | 0.17 ± 0.80 | |  |
|  | A3L1 | | | 39.1 ± 27.4 | | 48.4 | | 80.3 ± 0.45 | | | 34.5 ± 15.4 | 3.56 ± 3.49 | |  |
|  | A3L2 | | | 60.1 ± 29.4 | | 48.4 | | 80.7 ± 0.83 | | | 54.1 ± 19.6 | 1.78 ± 2.32 | |  |
|  | A3L3 | | | **79.0 ± 13.4** | | 48.4 | | 81.2 ± 0.94 | | | 70.5 ± 15.7 | 0.24 ± 0.72 | |  |
| Optimum for fishery rebuilding under considerable overfishing | None | | | NA | | NA | | NA | | | NA | NA | |  |
|  | A1 | | | 51.4 ± 6.58 | | 14.5 | | 11.1 ± 12.0 | | | 77.9 ± 11.5 | 1.63 ± 4.20 | |  |
|  | A2 | | | 49.3 ± 13.4 | | 22.5 | | 33.8 ± 24.0 | | | 55.1 ± 13.5 | 3.78 ± 6.26 | |  |
|  | A3 | | | **60.2 ± 8.56** | | 27.8 | | 46.4 ± 14.1 | | | 38.4 ± 13.0 | 1.16 ± 3.39 | |  |
|  | L1 | | | 30.6 ± 10.8 | | 27.1 | | 55.1 ± 11.4 | | | 98.7 ± 1.52 | 2.12 ± 3.64 | |  |
|  | L2 | | | **18.2 ± 9.74** | | 87.1 | | 84.3 ± 7.52 | | | 93.7 ± 4.60 | 6.75 ± 6.16 | |  |
|  | L3 | | | 24.7 ± 9.41 | | 100 | | 97.3 ± 2.84 | | | 82.9 ± 9.26 | 6.46 ± 5.96 | |  |
|  | A1L1 | | | 30.7 ± 13.6 | | 35.9 | | 62.9 ± 14.5 | | | 75.6 ± 12.3 | 4.21 ± 6.04 | |  |
|  | A1L2 | | | 24.9 ± 10.4 | | 92.1 | | 85.8 ± 7.29 | | | 73.0 ± 11.1 | 6.34 ± 6.32 | |  |
|  | A1L3 | | | 32.0 ± 9.73 | | 100 | | 97.5 ± 2.67 | | | 68.7 ± 10.1 | 5.00 ± 5.65 | |  |
|  | A2L1 | | | 37.7 ± 14.4 | | 46.2 | | 69.4 ± 13.8 | | | 55.8 ± 14.4 | 4.58 ± 6.14 | |  |
|  | A2L2 | | | 38.1 ± 13.0 | | 90.6 | | 87.0 ± 6.49 | | | 57.4 ± 14.1 | 4.26 ± 5.45 | |  |
|  | A2L3 | | | 43.2 ± 12.8 | | 100 | | 97.8 ± 2.39 | | | 58.1 ± 14.1 | 2.88 ± 4.34 | |  |
|  | A3L1 | | | 56.7 ± 13.4 | | 49.2 | | 72.6 ± 9.65 | | | 43.8 ± 15.8 | 1.60 ± 3.72 | |  |
|  | A3L2 | | | 55.8 ± 15.1 | | 87.5 | | 88.8 ± 5.18 | | | 48.7 ± 17.0 | 1.48 ± 3.03 | |  |
|  | A3L3 | | | 54.1 ± 16.2 | | 100 | | 98.2 ± 1.96 | | | 50.1 ± 17.4 | 1.33 ± 2.30 | |  |
| Maximum without fisheries costs under heavy overfishing | | All scenarios | | NA | | NA | | NA | | NA | | NA | | |
| Optimum for fishery rebuilding under heavy overfishing | | None | | NA | | NA | | NA | | NA | | NA | | |
|  |  | A1 | | 46.6 ± 14.6 | | 72.4 | | 20.1 ± 16.3 | | 77.3 ± 11.4 | | 0.87 ± 2.05 | | |
|  |  | A2 | | 54.0 ± 13.2 | | 88.6 | | 39.5 ± 16.8 | | 57.0 ± 13.7 | | 0.82 ± 2.07 | | |
|  |  | A3 | | **67.6 ± 8.86** | | 94.4 | | 56.7 ± 14.2 | | 44.5 ± 14.1 | | 0.13 ± 0.62 | | |
|  | | L1 | | 30.8 ± 12.3 | | 88 | | 50.4 ± 13.3 | | 98.2 ± 1.56 | | 1.44 ± 2.41 | | |
|  | | L2 | | **28.2 ± 7.90** | | 100 | | 79.0 ± 7.80 | | 94.3 ± 3.98 | | 2.15 ± 3.30 | | |
|  | | L3 | | 29.7 ± 9.46 | | 100 | | 96.6 ± 3.36 | | 83.3 ± 9.02 | | 2.17 ± 3.49 | | |
|  | | A1L1 | | 38.5 ± 11.5 | | 96.3 | | 57.8 ± 12.4 | | 76.4 ± 11.5 | | 1.41 ± 2.69 | | |
|  | | A1L2 | | 37.0 ± 8.36 | | 100 | | 81.7 ± 7.13 | | 75.2 ± 11.5 | | 1.53 ± 2.94 | | |
|  | | A1L3 | | 37.0 ± 11.1 | | 100 | | 97.1 ± 2.95 | | 69.3 ± 11.4 | | 1.48 ± 2.88 | | |
|  | | A2L1 | | 51.6 ± 11.1 | | 98.3 | | 65.5 ± 10.4 | | 60.1 ± 15.2 | | 0.71 ± 1.89 | | |
|  | | A2L2 | | 48.4 ± 12.2 | | 100 | | 84.9 ± 6.05 | | 60.6 ± 16.0 | | 0.69 ± 1.78 | | |
|  | | A2L3 | | 45.9 ± 14.4 | | 100 | | 97.6 ± 2.46 | | 57.8 ± 16.2 | | 0.75 ± 1.73 | | |
|  | | A3L1 | | 63.5 ± 11.6 | | 99.5 | | 74.1 ± 8.92 | | 48.7 ± 17.1 | | 0.13 ± 0.55 | | |
|  | | A3L2 | | 58.1 ± 14.4 | | 100 | | 88.5 ± 5.11 | | 49.3 ± 18.6 | | 0.22 ± 0.70 | | |
|  | | A3L3 | | 54.1 ± 16.3 | | 100 | | 98.2 ± 1.96 | | 48.4 ± 19.2 | | 0.32 ± 0.85 | | |
