# Supplementary material for: Marine Reserve Targets to Sustain and Rebuild Unregulated Fisheries
Source: PLoS Biol. 2017 Jan 5;15(1):e2000537. doi: 10.1371/journal.pbio.2000537 (PMC5215937; doi:10.1371/journal.pbio.2000537)
Supplement: S3 Table — The numbers in brackets denote default assumptions (if any) and samples sizes presented in Fig 1. The two Keppel islands scenarios were run using both the spatially-implicit and spatially-explicit version of our model. (DOCX) [file pbio.2000537.s013.docx]

| Parameters | Generic | Keppel islands 1 (K1)  (*Lutjanus carponotatus*) | Keppel islands 2 (K2)  (*Plectropomus maculatus*) |
| --- | --- | --- | --- |
| Fishing pressure (× *F*_MSY_) | 1-2 (n = 11) | 1-2 (n = 11) | 1-2 (n = 11) |
| Annual survival (*s*) | 0.18-0.88 (n = 139) | 0.46 (n = 1) | 0.66 (n = 1) |
| Brody growth coefficient (*p*) | 0.04-4.92 (n = 139) | 0.37 (n = 1) | 0.21 (n = 1) |
| Exchange of larvae (*d*) | 0-100% (n = 11) | Simulated (see S4 Fig for spatially-implicit analysis) | Simulated (see S4 Fig for spatially-implicit analysis) |
| Exchange of adults (*m*) | 0-100% (n = 11) |  |  |
| Compensation (*h*) | 0.5-0.9 (0.7, n = 5) | 0.5-0.9 (0.7, n = 5) | 0.5-0.9 (0.7, n = 5) |
| Reserve sizes (mean ± SD) | Implicit | 1-20 km (4 ± 4 km) | 1-20 km (4 ± 4 km) |
| Fisher distribution | Stationary | Stationary | Stationary |
